# Plant-mediated green nanoparticles: combining nanometal and biometabolite potential for Alzheimer’s treatment

**DOI:** 10.1186/s12938-026-01572-z

**Published:** 2026-05-28

**Authors:** Farahnaz Behzad, Foad Rahmanpour Leili, MohammadJavad Ebrahimi, Maral Moafi, Pourya Bagherian kenari, Ali Alizadeh, Soheila Adeli, Masood Khosravani, Mohammad Amin Jadidi Kouhbanani, Javad Fahanik-babaei

**Affiliations:** 1 Department of Medical Nanotechnology, School of Advanced Technologies in Medicine, Tehran University of Medical Sciences, Tehran, Iran; 2 Electrophysiology Research Center, Neuroscience Institute, Tehran University of Medical Sciences, Tehran, Iran; 3 MD, Faculty of Medicine, Department of Neurology, Tonekabon Branch, Islamic Azad University, Tonekabon, Iran; 4 Cell Biology and Anatomical Sciences, School of Medicine, Shahid Beheshti University of Medical Sciences, Tehran, Iran; 5 School of Medicine, Babol University of Medical Sciences, Babol, Iran; 6 Department of Medical Physics and Biomedical Engineering, School of Medicine, Tehran University of Medical Sciences, Tehran, Iran; 7 Department of Pharmaceutical Biotechnology, Faculty of Pharmacy, Tehran University of Medical Sciences, Tehran, Iran

**Keywords:** Alzheimer’s disease, Acetylcholinesterase inhibitors, Plant metabolites, Green synthesis, Metal nanoparticles

## Abstract

Alzheimer’s disease (AD) can cause cognitive and memory dysfunction due to insufficient acetylcholine (ACh). In principle, acetylcholinesterase (AChE) hydrolyzes ACh into acetic acid and choline, rendering the latter inactive. Acetylcholinesterase inhibitors (AChEI) are currently the main treatment strategy used to increase ACh availability and decrease the effects of cholinergic loss. The FDA has currently approved donepezil, rivastigmine, and galantamine (GAL) as AchEI drugs to treat AD. Among these drugs, only the alkaloid galantamine is found naturally among the AChEIs. The synergistic effect of plant metabolites and metal nanoparticles (MNPs) presents a promising avenue for AD treatment. Green synthesis approach leverages the unique properties of MNPs combined with the therapeutic potential of plant-derived compounds. In the present review, recent developments in nanotechnology, including the green synthesis of MNPs, have been discussed in relation to the treatment of AD. Given the recent advancements, we hope that the combination of nanotechnology and medicinal plants will eventually result in the development of highly successful strategies for the treatment of AD.

**Graphical Abstract:**

**Figure:**
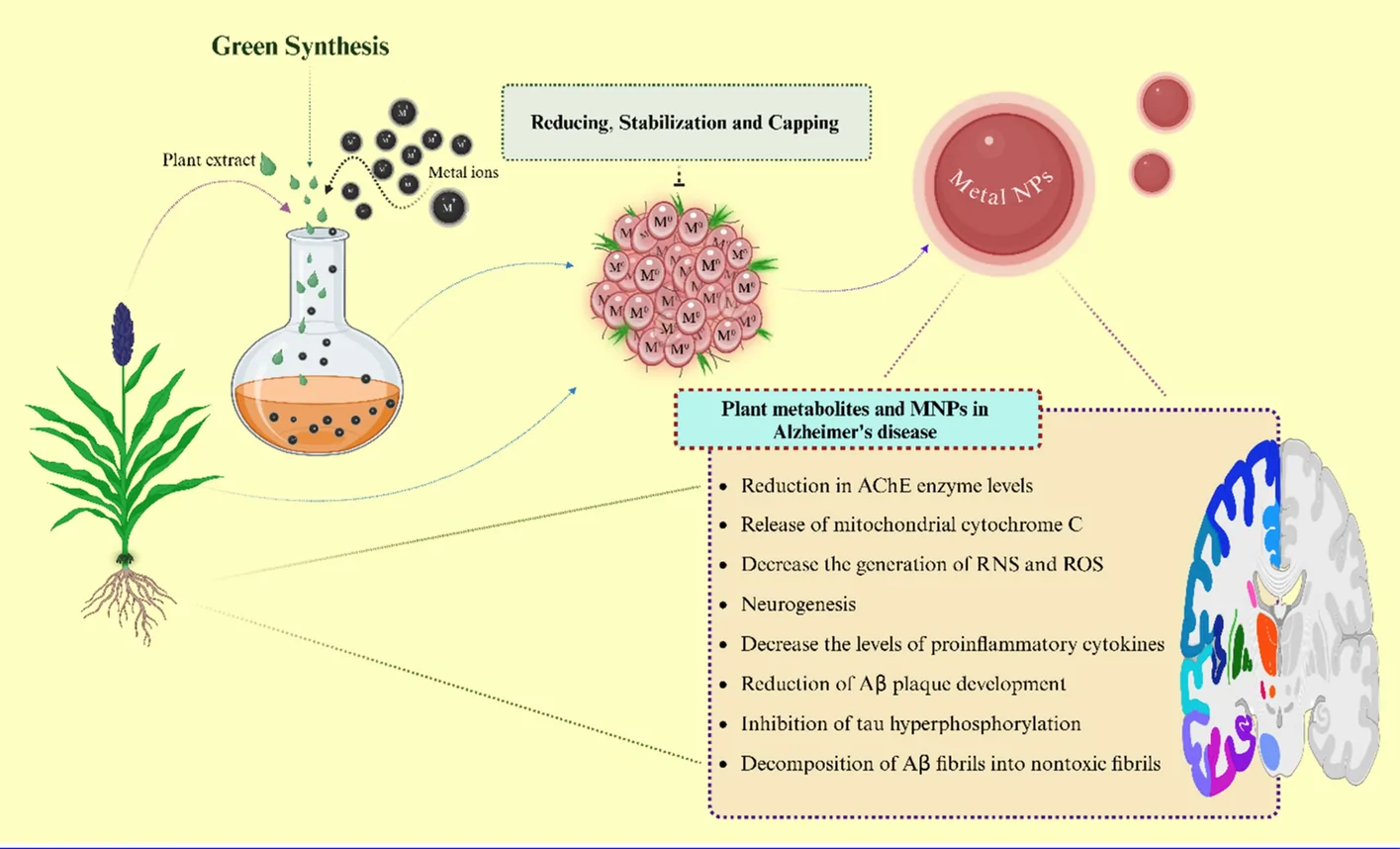
Created in BioRender. Jadidi Kouhbanani, M. A. (2026) https://BioRender.com/e45n589

## Introduction

The main neurotransmitter of the brain is acetylcholine (ACh), whose absence can lead to the occurrence of Alzheimer’s disease (AD) [1–3]. The ACh molecule acts as a neurotransmitter in the synaptic gap and provides information flowing among neurons, so the deficiency of ACh and the loss of the cholinergic system explain the cholinergic hypothesis [4, 5]. Acetylcholinesterase (AChE) (EC 3.1.1.7) can be the predominant marker of cholinergic system deficiency, which degrades ACh and/or cholineacetyltransferase inhibition, which is involved in the synthesis of ACh [6]. By inhibiting AChE with the help of inhibitors, ACh can be recovered. To address this issue, numerous studies on AChE inhibition have been conducted. ACh levels in several parts of the brain can be raised by inhibiting the AChE, and symptoms linked to AD’s progressive loss of cholinergic function can be alleviated [7].

Many drugs approved by the Food and Drug Administration https://www.fda.gov/ (FDA) to treat AD are available on the market such as galantamine (Razadyne^®^ or Reminyl^®^), rivastigmine (Exelon^®^), and donepezil (Aricept^®^) [8, 9] as AChE inhibitors. AChE inhibitors are actually the only class of drugs that have a certain success rate when used to treat AD, but their use has been restricted because of their side effects, which include upset stomach, nausea, vomiting, and diarrhea [10, 11].

Recent advancements at the nexus of nanotechnology and green chemistry suggest substantial promise in the biomedical sciences [12–17]. Other chemical and physical synthesis methods are often discouraged due to their inherent disadvantages, which include cost and toxicity [18–20]. Furthermore, a recent study revealed that certain chemicals used in the synthesis of NPs may stay bonded to their surface, making them unsuitable for use in biomedical applications. Consequently, great interest has been growing over the past several years to fabricate materials of interest using environmentally friendly methods [21–24]. Typically, using pure phytochemicals or medicinal plants with potential medical benefits is a part of the green chemistry-based approach [25–27]. According to reports, the green synthesis of NPs involves a wide range of molecules, including proteins, low molecular weight compounds like terpenoids, alkaloids, amino acids, alcoholic compounds, glutathiones, polysaccharides, antioxidants, organic acids (protocatechuic acid, tartaric, ascorbic, malic, and oxalic), quinones, and polyphenols (taxifolin, flavones, catechin, procyanidins of various chain lengths formed by catechin and epicatechin units, and phenolic acids) [28].

According to studies, medicinal plants may help AD patients’ memory and cognitive function, and combining nanotechnology and plant extracts increases therapeutic efficacy [29–31]. Gold nanoparticles (AuNPs) have shown promising strides in combating neurodegenerative ailments like AD by inhibiting amyloid *β* (Aβ) protein aggregation, a hallmark of the disease [29, 30, 32]. For instance, *Terminalia arjuna* a medicinal plant used in folklore medicines for a variety of health purposes, was used in a study for the biogenic synthesis of AuNPs. In addition to effectively inhibiting Aβ fibrillation, the generated AuNPs also caused the mature fibrils to become destabilized. Additionally, the ChE was markedly inhibited by the AuNPs. The *Terminalia arjuna* mediated AuNPs were discovered to be nontoxic and biocompatible while being studied for toxicity in zebra fish [33]. Also, Silver nanoparticles (AgNPs) exhibit attractive properties for AD treatment due to their multifaceted therapeutic potential. AgNPs have shown significant inhibitory activity against Aβ protein aggregation [34, 35]. Additionally, Au/Ag NPs have demonstrated inhibitory effects against AChE and reactive oxygen species (ROS), crucial factors in neurodegenerative disorders, with an inhibition rate of ∼50% against esterase and ∼75% against ROS [36]. *Kickxia elatine* extract and synthesized AgNPs-*Kickxia elatine* will bear effective anti-AD activity because *Kickxia elatine* contains biologically active components [37]. *Scoparia dulcis L* extract could lead to the production of AgNPs with strong antioxidant potential, which would make them useful for a range of bioapplications, including possible AChE inhibition research [38]. Also, the green zinc oxide NPs (ZnO NPs) [39] and iron oxide NPs (IONPs) [40] formulations are helpful in treating neurological illnesses like AD.

In this review, we first provide an overview of AD, and then we discuss phytochemicals and natural bioactive compounds in AD. We show that plant-derived secondary metabolites influence the size, shape, stability, and bioactivity of green-synthesized MNPs, and, for the first time, we analyze how retention or removal of capping agents is related to MNPs properties and behavior, highlighting the synergistic integration of plant metabolites and MNPs via green synthesis and their potential to enhance efficacy in AD.

## Alzheimer’s disease

The main sign of Alzheimer’s disease (AD), a neurological one, is memory loss. The precise cause of AD is unknown, although it has existed for millions of centuries. AD-like pathophysiology is developed by physiological abnormalities or alterations, including hyperphosphorylation of tau protein, extracellular Aβ generation, oxidative stress, mitochondrial dysfunction, cholinergic imbalance, and risk factors, including environmental factors, aging, and mutations in genes [41]. Amyloid plates, neurotransmitter disruption, and inflammatory mechanisms are the key clinical symptoms of the central nervous system. A gradual loss of neurons in the basal forebrain contributes to cholinergic hippocampal innervations; these alterations can impair mental function and cause neurological conditions as well as memory loss [42–44].

### Risk factors of Alzheimer’s disease

**Fig. 1:**
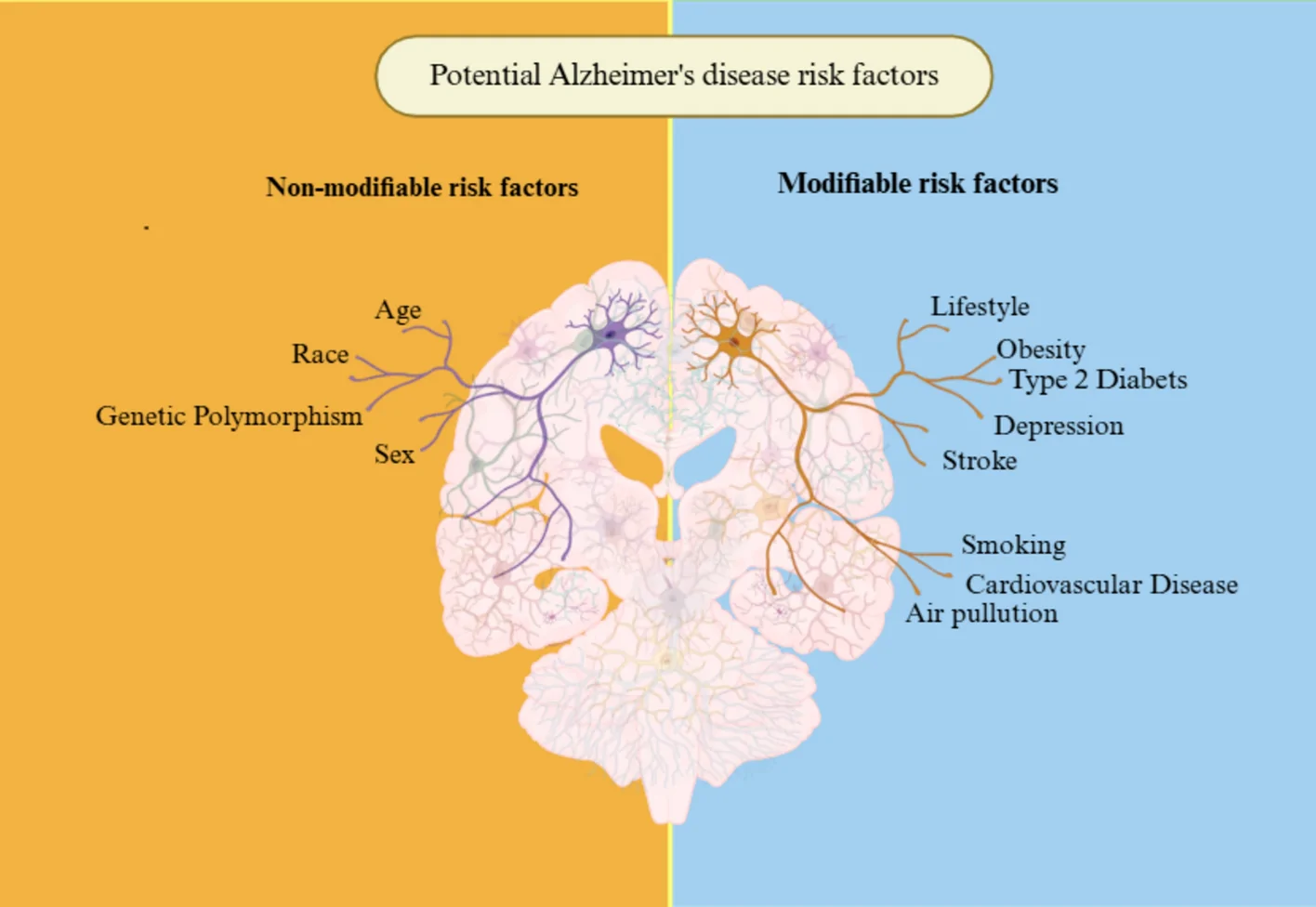
Potential Alzheimer’s disease risk factors. Modifiable and non-modifiable risk factors are the two types of risk factors for AD. Created in BioRender. Jadidi Kouhbanani, M. A.(2026) https://BioRender.com/c94r423

Several factors may contribute to the development of AD, though the precise cause is unknown and may have multiple origins. In recent years, factors influencing the risk of growing AD have been identified, including lifestyle and genetic, environmental, and pathophysiological factors. However, the most significant risk factor is age, as the likelihood of developing AD increases with age [45, 46]. Factors influencing the risk of AD development are shown in Fig. 1.

#### Lifestyle and health factors

According to an organized study, psychosocial factors and actively integrated lifestyles over time may lower the risk of AD and dementia [47]. Obesity, diabetes, high blood pressure, and high cholesterol can all increase the risk of developing AD [48]. People should lead healthy lives in order to prevent these risk factors. For instance, low-intensity exercises like walking can also reduce the risk of mental illnesses and AD [49]. Also, the diet which includes fish oil containing rich docosahexaenoic acid (DHA) and polyunsaturated fatty acid (PUFA) helps to reduce the prevalence of AD [50, 51]. Simons et al., reviewed the correlation of AD with cholesterol level in the body; that is, the cholesterol level in the body increases as the age increases. To decrease cholesterol level, statins are prescribed. In these patients, Aβ production and secretion were reduced drastically along with cholesterol levels, which is due to inhibition of de novo synthesis by statins [52, 53]. On the other hand, participating in complex work may reduce the risk of AD and vascular dementia, as per the Canadian Study of Health and Aging [54].

#### Environmental factors

Long-term exposure to a number of common environmental risk factors, such as heavy metal, air pollutants, pesticides, and industrial chemicals, has been connected to an elevated risk of AD disease and a progressive decline in mental functioning. There is growing evidence linking the development of AD to pollutants and fungi. They are highly oxidative and associated with apoptotic signaling, unfolded protein accumulation, calcium homeostasis, and mitochondrial dysfunction—all of which are recognized characteristics of AD [55].

#### Genetic factors

Early onset of autosomal dominant familial AD has been linked to mutations in the amyloid precursor protein (APP) gene on chromosome 21 or the presenilin 1 or 2 (PREN 1 or 2) genes on chromosomes 1 and 14 [56, 57]. Also, the age at which AD begins to develop decreases in a dose dependent manner, and the risk of AD increases as the number of apolipoprotein E-4 (APOE-4) alleles increases. The risk effect of the APOE-4 allele on AD decreases with age, and the 4 allele is known to be involved in 15–20% of AD cases. On the other hand, mutations on the Apo 2 gene, which is essential for maintaining the equilibrium between Aβ peptide deposition and clearance, are associated with a high risk of developing sporadic AD [58, 59]. Amyloid and microtubule-associated tau protein are undoubtedly the most well-studied proteins linked to the pathogenesis of AD [60]. It is not surprising that the pathological diagnosis of AD is based on both amyloid and protein deposits [61].

### Epidemiology of Alzheimer’s disease

According to the epidemiological data available, is predicted to affected persons will double every 20 years, reaching 81.1 million by 2040 [46, 62]. In the Middle East and North Africa, the Age-adjusted point prevalence of dementia was 777.6 per 100,000 in 2019, a 3% increase since 1990, according to Safiri et al. [63]. This pattern is similar to the rise in dementia prevalence seen worldwide, which is mostly caused by population aging and longer life expectancies. Age-standardized prevalence rates were among the lowest in the United Arab Emirates and among the highest in Bahrain and the United Arab Emirates [64]. About 55 million people worldwide, including 6.9 million Americans aged 65 and over, were estimated by the Alzheimer’s Association to have AD or another form of dementia in 2024. By 2060, there might be 13.8 million Americans with AD if no progress is achieved in the prevention or treatment of the disease [65]. Of them, 73% are 75 years of age or older. About one in nine people aged 65 and older (10.9%) has AD. Women make up nearly two-thirds of AD patients in the United States. Approximately 200,000 individuals with AD are younger than 65, but the majority (81%) are older than 75 [65]. However, over one-third of extremely old individuals (85 + years old) may show signs and symptoms of dementia, and around one in ten older persons (65 + years old) in affluent nations suffer from dementia to some degree (Bright Focus Foundation, 2025). According to estimates, 7.2 million Americans aged 65 and older will have Alzheimer's dementia in 2025, and roughly 2.5 million of those affected will be 85 years of age or older, making up 33% of all Alzheimer’s dementia patients. According to the Alzheimer’s Association, 6.7 million persons aged 85 and older are predicted to develop Alzheimer's dementia by 2060, making up roughly half (48%) of all those aged 65 and older who have the disease. However, over one-third of extremely old individuals (85 + years old) may show signs and symptoms of dementia, and around one in ten older persons (65 + years old) in affluent nations suffer from dementia to some degree (BrightFocus Foundation, 2025). According to estimates, 7.2 million Americans 65 and older will have Alzheimer’s dementia in 2025, and roughly 2.5 million of those affected will be 85 years of age or older, making up 33% of all Alzheimer’s dementia patients. According to the Alzheimer’s Association, 6.7 million persons 85 and older are predicted to develop Alzheimer’s dementia by 2060, making up roughly half (48%) of all those 65 and older who have the disease.

According to the pooled data of European population-based research, the age-standardized prevalence of dementia and AD in people 65 + years old is 6.4% and 4.4%, respectively. In general, the global prevalence of dementia in those aged 60 and over was estimated to be 3.9% globally, with regional prevalences of 6.4% in North America, 5.4% in Western Europe, 4.6% in Latin America, 4.0% in China and Western Pacific regions, and 1.6% in Africa [65–67].

According to recent reports from the US Centers for Disease Control and Prevention and the UN Aging Program, the number of people aged 65 and older worldwide is predicted to rise from 420 million in 2000 to almost 1 billion by 2030, raising the proportion of older people from 7 to 12%. The number of people at risk will rise along with the global population’s continued aging, especially among the elderly. According to reports, in recent years, the mortality rate from AD has doubled. The risk of dying before turning 80 is twice as high for seniors with AD as for those without the condition. As a result, it is expected that this dementing illness will present significant obstacles to the public health and senior care systems in all countries across the world [46, 62, 66].

### Pathophysiology of Alzheimer’s disease

AD is a prevalent, useful, and intermittent degenerative brain disease. It is a gradual, widespread atrophy of the cerebrum rather than merely a reduction in brain weight and volume. According to the pathological testing, this disorder is characterized by a substantial loss of neuronal cells, particularly in the hippocampus and medial temporal lobes [68].

The development of neurofibrillary tangles (NFTs), which are made up of an insoluble intracellular hyperphosphorylated microtubule-associated protein tau, and the presence of increased extracellular Aβ plaques, which are caused by the aggregation and poor clearance of Aβ oligomers (hydrophobic Aβ aggregates), are the two primary pathological characteristics of AD [69]. Neurons, especially cholinergic neurons in the neocortex and basal forebrain, are lost in conjunction with these pathological alterations. In the following, AD-related hypotheses will be discussed in detail [70].

#### Theories of Alzheimer’s disease

##### Amyloid beta (Aβ) and Tau protein

Current research on AD neuropathology indicates that intracellular tubulin associated unit (tau) neurofibrillary tangles and extracellular amyloid plaques are the main histopathologic lesions of AD [70]. Numerous investigations were carried out to suggest various mechanisms, such as the “amyloid cascade hypothesis”, that contribute to the complexity of AD pathology. The extracellular accumulation of Aβ peptide forms amyloid plaques which hinders cell to cell communication of neurons and leads to synaptic and cell death [71]. In this case, there is an imbalance between production, clearance, and Aβ aggregation [72].

**Fig. 2:**
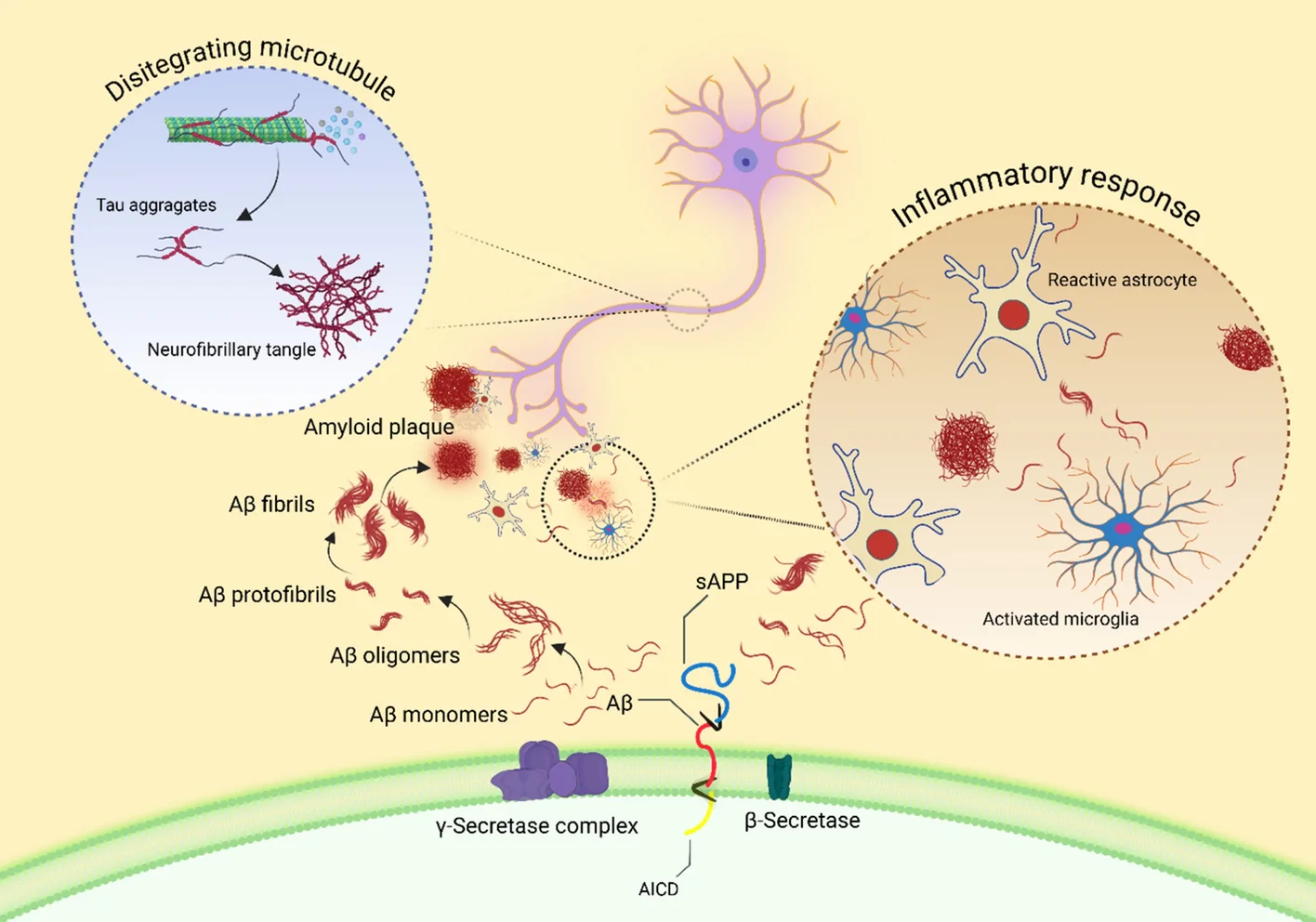
An overview of the major hallmark events leading to Alzheimer’s disease: Amyloid beta and Tau protein. Abbreviation: sAPP: soluble amyloid precursor protein; AICD: APP intracellular domain; Aβ: Amyloid beta. Created in BioRender. Jadidi Kouhbanani, M. A. (2026) https://BioRender.com/f01p947

The creation of NFTs due to the hyper-phosphorylation of “Tau”, a protein component of microtubules, and as a result of the imbalance between the formation and clearance of Aβ is another hypothesis regarding AD. The tau protein, which is a crucial component of the neuronal cytoskeleton and has a molecular weight varying between 45 kilodalton (KD) to 70 KD, promotes the assembly of microtubules in healthy brain tissue. The brain cell structure is supported by microtubules, here tau protein binds and stabilizes axonal microtubules. The tau protein’s ability to stabilize microtubules is regulated by phosphatases and kinases. The dysfunction of Tau in AD leads to the accumulation of NFTs inside neurons, which restricts the transport of nutrients and mitochondria, and disrupts the growing of new neurons [73–75] (Fig. 2). In the brain, excitotoxicity from all of these automatic processes results in neuron dysfunction, neurotoxicity, and death [75, 76].

###### APP cleavage and Aβ generation

In healthy human beings, amyloid precursor proteins (APP) are broken into Aβ peptide and fragments of soluble amyloid precursor protein (sAPP), which gets eliminated in the future. In typical physiological conditions, APP, a transmembrane protein with three domains one outside the cell, one in the membrane of cells, and one inside the cell has been thought to be essential for cell migration, signal transduction, and axonal elongation [77]. Aβ is a 4 kDa fragment of APP, a bigger precursor molecule that is generated by blood and vascular cells (such as platelets), and brain neurons, to a lower extent, astrocytes. The brain’s dysregulation of Aβ levels results in the deposition of Aβ and the development of senile plaque, which impairs cognition in AD patients [78–80]. Two enzymes, namely *γ*-secretase and *β*-secretase (BACE1), then break down the APP, producing Aβ-peptides, the most common of which are Aβ40 and Aβ42 (Fig. 2). The number of residues in these two main Aβ isoforms varies; Aβ42 contains two more residues at its C-terminus than Aβ40. It has been demonstrated that the C terminus of APP is essential for both neuron survival and the expression of specific genes. The Aβ isoform that forms amyloid plaques in AD is mostly Aβ42, and some plaques only contain the Aβ42 isoform [80–82].

**Fig. 3:**
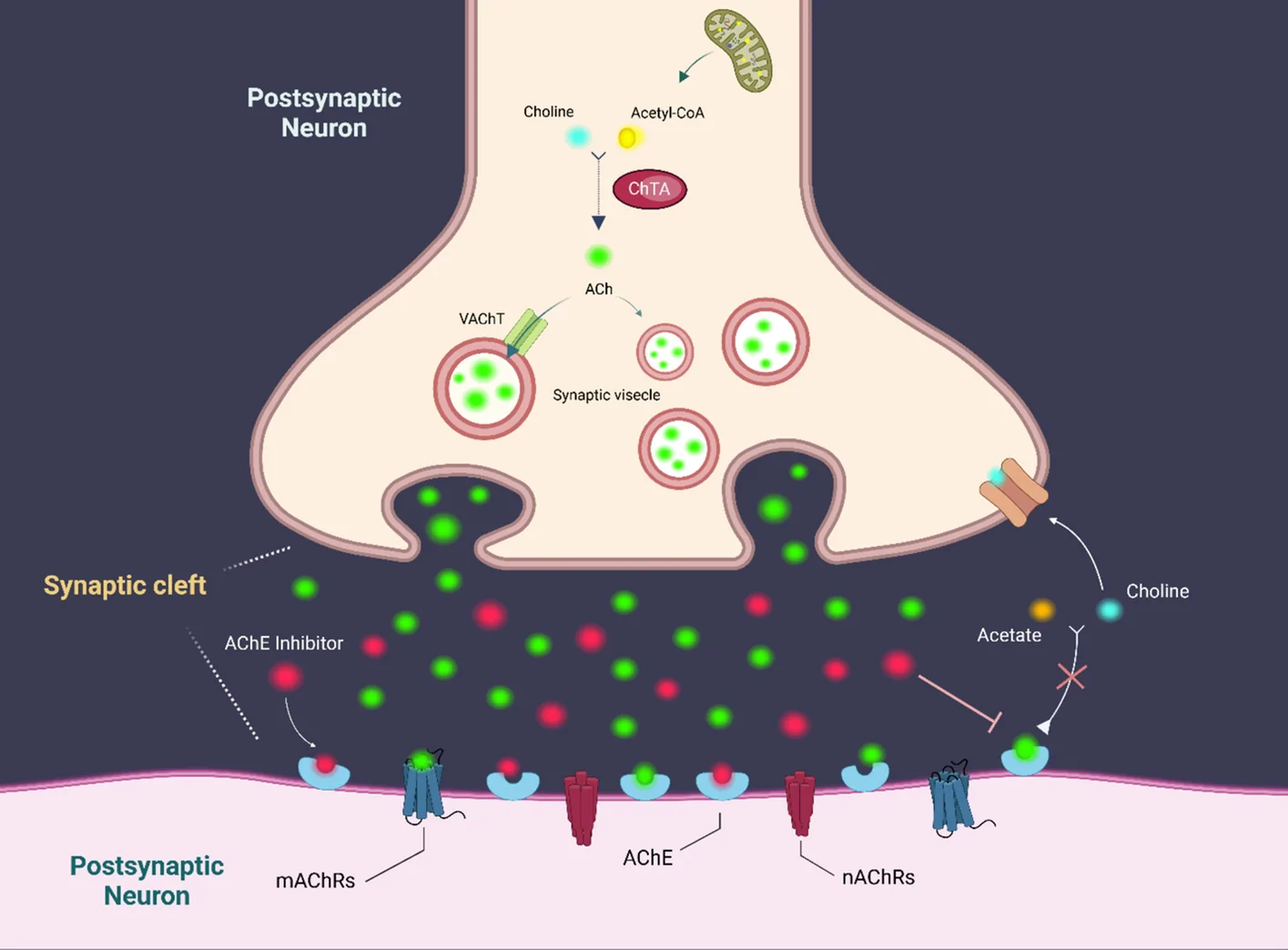
The summary of the mechanisms of action of acetylcholinesterase inhibitors. Abbreviation: AChE: Acetylcholinesterase; ChTA: choline acetyltransferase; Ach: acetylcholine; VAChT: Vesicular acetylcholine transporterVAChT; mAChRs: Muscarinic acetylcholine receptors; MAChRs: Nicotinic acetylcholine receptors (nAChRs). Created in BioRender. Jadidi Kouhbanani, M. A. (2026) https://BioRender.com/x96b477

##### Cholinergic hypothesis

The enzyme choline acetyltransferase, which is in charge of the production of Ach, was linked in the 1970s to neocortical and presynaptic cholinergic deficiencies. The cholinergic hypothesis of AD was put out because of the critical role that ACh plays in cognitive function [83]. Certain parts of the cerebral cortex exhibit decreased activity, according to experimental studies using the brains of AD patients as a model. It is feasible to mention the choline acetyltransferase activity in this context [84, 85]. Remarkably, an in vitro investigation shows that Aβ peptide causes the suppression of cholinergic neurotransmission [85–87]. The research has shown that the neurotoxicity of Aβ oligomers and the interactions between AChE and Aβ peptide are linked to cholinergic synaptic loss and the production of amyloid fibrils [88–90]. In addition, other studies showed that cognitive function is reduced when the amount of nicotinic and muscarinic ACh receptors in presynaptic cholinergic portions is reduced [85]. It should be emphasized that the primary treatment target in the cholinergic hypothesis is the AChE enzyme [85].

##### Metal ion hypothesis

Numerous researches have looked into the possible connection between AD and biometals [91, 92]. Biometals are metal ions that have a useful role in living things, whereas other metal ions are harmful or inert. Any metal ion’s dyshomeostasis in the body often results in illness [92]. For instance, there is proof that AD brains exhibit biometal dyshomeostasis. Zinc was associated with Aβ in the first study to assess biometals and Aβ, published by Bush et al., in 1994 [91]. Zn is essential for neuronal signaling and neurotransmission, and neurological conditions like AD have been connected to the metal's build-up in the brain. Zn-treated cells had a markedly higher AChE activity. Treatment with zinc dramatically decreased glutathione levels and catalase and superoxide dismutase activity [2, 93]. Aβ directly coordinates other biometals, particularly Cu, and biometals like iron can accumulate to high concentrations (~ 1 mM) in plaques [92, 94, 95].

Iron dyshomeostasis, a disruption of iron balance, is one of the primary causes of AD neuronal death [96]. All things considered, pathogenic protein deposition is significantly influenced by the build-up of biometals in Aβ plaques and NFTs. For example, they could interfere with clearance processes, modify the activity of vital enzymes, or change the shape of proteins. Metals that are trapped in protein deposits have the potential to increase toxicity by starting a chain reaction that produces ROS [97].

##### Oxidative stress hypothesis

The body creates reactive nitrogen species, ROS, and other very reactive and unstable compounds during normal metabolic activities. An effective antioxidant defense system typically maintains these chemicals at low levels to shield cells from oxidative harm [97–99]. The state of cellular damage brought on by free radicals due to the antioxidant system’s inadequate performance is known as oxidative stress. The pathophysiology of AD is closely linked to oxidative stress, Aβ deposition, tau hyperphosphorylation, cross-linking of nerve fibers, and nerve cell damage, all of which can be brought on by an imbalance of metal ions (such as Zn, iron, calcium, Cu, and others) [97, 100]. Thus, the development of NDs like AD is significantly influenced by oxidative stress. The majority of biomolecules are capable of oxidation, and in AD, the majority of these biomolecules originate from the neuronal membrane, where proteins, fatty acids, and lipids are oxidized [101, 102].

## FDA-approved treatments for Alzheimer’s

AChE inhibitors are currently the most frequently used therapy for AD. There are drugs on the market that have been approved by the FDA for the treatment of AD, including galantamine (Razadyne^®^ or Reminyl^®^), rivastigmine (Exelon^®^), and donepezil (Aricept^®^), which are AChE blockers [75, 103–106]. Table 1 lists the drugs approved by the FDA and also the pharmacological properties of the AChE inhibitors currently used to treat AD.

**Table 1:** FDA-approved anti-Alzheimer’s drugs and the pharmacological characteristics of the cholinesterase inhibitors [75, 103–106]. Created in BioRender. Jadidi Kouhbanani, M. A. (2026) https://BioRender.com/q83p142

Drugs and chemical structures	Brand name	Biosynthesized	Chemical class	Target enzymes	Recommended dose	Available formulations
	Razadyne® or Reminyl®	The only molecule among the AChE inhibitors, which is naturally found	Phenanthrene alkaloid	AChE	8 mg/day for 4 weeks (with meal), then titrate to 24 mg/day (twice daily)	Oral solution and tablet
	Aricept^®^	–	Piperidine	AChE	5 mg/day for 4–6 weeks, titrate to 10 mg/day (once daily)	Tablet
	Exelon^®^	–	Carbamate	BuChE and AChE	12 mg for 2–4 weeks (twice a day)	Oral solution and capsule
	Cognex^®^	–	Acridinamide	BuChE and AChE	80–160 mg/day (four times daily)	Capsule
	Namenda^™^	–	of adamantanes and a primary aliphatic amine	NMDA receptor agonist	28 mg/day	–

The cholinergic hypothesis suggests that ACh, which is hydrolyzed by AchE, is important in AD pathology; therefore, the basic cause of AD is a decrease in ACh production [2]. The enzyme AChE is responsible for cholinergic synaptic connection’s neurotransmission termination. This enzyme accelerates ACh hydrolysis. Lower levels of the neurotransmitter ACh and the degree of cognitive impairment have been related to the reduction of cholinergic activity caused by AChE. By preventing ACh turnover and raising synaptic levels of this neurotransmitter, these drugs make up for the loss of cholinergic neurons and provide symptomatic relief [107, 108]. After binding to AChE, ACh is broken down into choline and acetate, thereby stopping its neurotransmitter function. Additionally, even at low ACh concentrations, the enzyme exhibits enhanced catalytic activity [7, 109].

AChE may have a pathological function in AD by affecting the process that leads to amyloid toxicity and the onset of AD. This is because when AChE is incorporated into AD amyloid aggregates, stable complexes are formed that alter the enzyme’s biochemical and pharmacological characteristics and increase the neurotoxicity of the Aβ fibrils. Currently, the most successful treatment for AD is acetylcholinesterase inhibition (AChEI). ACh levels in various brain regions can be raised by inhibiting the cholinesterase enzyme (AChE), and the symptoms linked to progressive decrease in cholinergic function improve [75, 109–111] (Fig. 3).

Rivastigmine: The reversible cholinesterase inhibitor RVT ((S)-3-[1-(dimethylamino) ethyl] phenyl-N-ethyl-N-methylcarbamate, Table 1) aims to treat the symptoms of moderate to severe stages of AD. The fact that RVT has few drug interactions is important because it is intended for elderly patients, who often have comorbidities and utilize various medications. Based on reports, RVT did not interact with fluoxetine, diazepam, digoxin, warfarin, calcium channel blockers, or antihistamines [112, 113]. It is specific for the G1 isoform of AChE and is a gradually reversible dual inhibitor of acetyl and butyryl ChE (BuChE), which is not metabolized by the CYP450 system in the hepatic system. Because AChE and BuChE degrade RVT at the synapse, it is referred to as “pseudo-irreversible” [109, 114, 115]. When the drug is used orally, adverse effects include anorexia, dyspepsia, and vomiting. Nevertheless, these side effects can be managed with time and making the drug less unpleasant. Despite its unique characteristics, it has been associated with a higher incidence of side effects than other ACE inhibitors; nevertheless, regular dosing can lessen these problems [109, 116–118].

Galantamine: The only natural molecule found in AChE inhibitors is the alkaloid GAL, which has been approved by the FDA [109, 119], Table 1 (for a comperhensive explanation, see Sect. "The role of alkaloids in AD").

Donepezil: A derivative of indanonebenzylpiperidine, DZP is widely used to treat AD (Table 1). DZP is a ChE inhibitor that may help control AD symptoms by keeping acetylcholine from being broken down and enabling the brain to store more ACh. This may help prevent AD from getting worse. By binding to AchE and reversibly blocking Ach hydrolysis, DZP raises the amount of ACh at synapses [109, 120–122]. Cholinergic side effects are mild and temporary, and the treatment is generally accepted. DZP, a drug that doesn’t slow the progression of the disease, is used to treat symptoms of AD like enhanced behavior and cognition [120–122].

Tacrine: Tetrahydroacridine tacrine, marketed under the name Aricept (Table 1), was the first drug authorized by the USFDA to treat mild to moderate AD. In AD patients, tacrine (80–160 mg/day) improves behavioral impairments and cognitive performance. In neurological conditions such as AD, tacrine is a good AChE inhibitor. It is metabolized by the P-450IA2 isoenzyme, which may interact with other drugs metabolized by this enzyme. Bapropion and tramadol/acetaminophen can rarely induce seizures, but when combined with tacrine, they may cause seizures. Combining teriflunomide and leflunomide with tacrine may make liver damage more likely. Only slight cognitive improvements were seen, and it was unable to stop the disease’s progression [109, 123, 124]. Unfavorable damage to the liver occurred in almost one-third of those taking tacrine. To evaluate liver function, regular blood tests were necessary. In certain instances, stopping the drug was necessary. The liver damage was reversible if caught early. Due to the liver toxicity noted above, tacrine eventually lost favor as new medications, with comparable efficacy but no liver harm, were created. Consequently, the company ceased the production of tacrine, and the medication was withdrawn in 2013 [125–127]. Since then, non-hepatotoxic tacrins have been described in a number of publications, and significant efforts have been made to develop multi-targeted tacrins by fusing their ChE inhibition profile with the control of additional biological targets implicated in AD [109, 123, 128].

Memantine: Since the FDA approved memantine (Namenda^™^) (Table 1), it has become the first exciting drug for treating people with moderate to severe AD. It works by regulating glutamatergic failure through a novel mechanism of action that is a moderate affinity, and non-competitive, voltage-dependent N-methyl-d-aspartate (NMDA) receptor antagonist with rapid on/off kinetics It enhanced patients' performance in daily functioning, behavioral disorders, and cognition. Memantine is more effective than a placebo at improving behavioral disorders and cognitive functions, both when used alone and in combination with DZP. Being a distinct non-competitive NMDA antagonist, memantine unquestionably maintains its status as a traditional neurochemical that can be used either by itself or in combination with other AChE inhibitors, like DZP [83, 109, 129].

## Evolution of treatment strategies

The newest and most promising field of study for targeted delivery of drugs is the treatment of AD using nanomedicine and phytotherapeutics. Numerous phytochemicals have reportedly shown significant effectiveness in treating AD [130, 131]. The use of natural products-based nanotechnology (green synthesis) can increase the effectiveness. With green synthesis method, phytochemicals are used during the synthesis of NPs in place of hazardous chemicals as reducing and stabilizing agents [132–134]. In this section, the treatment strategies of AD start from MNPs (Sect. “Alzheimer’s disease and the role of metal nanoparticles”), then treatment through medicinal plants and their bioactive compounds is discussed in detail below (Sect. “The role of plants bioactive compounds in the treatment of AD”). Finally, the synergistic effect of plant metabolites and MNPs in the treatment of AD is discussed (Sect. “Green synthesis: combining the potential of plant metabolites and metal nanoparticles”).

### Alzheimer’s disease and the role of metal nanoparticles

MNPs are the most popular class of NPs, which can be defined by their distinct features and widespread use. MNP’s size, dispersity, shape, physical characteristics, and chemical properties all affect their applicability, and these factors are affected by the approach used for producing them. MNPs are primarily divided into two classes: noble MNPs, which include gold (Au), platinum (Pt), silver (Ag), and ruthenium (Ru); and magnetic MNPs, which include Nickel (Ni), titanium (Ti), copper (Cu), iron (Fe), cobalt (Co), and zinc (Zn) NPs. Some MNPs, such as Au, Ag, and Zn, have positive effects and can effectively treat AD. Following administration, the body and various organs, including the brain, easily absorb MNPs. In contrast to bulk metal, these MNPs' high surface area, size range of 1–200 nm, and distinct physicochemical properties enhance the rate of absorption, bioavailability, and biocompatibility [15, 35, 134–136].

The following is the thorough discussion of how these MNPs have a preventative impact on AD: MNPs provide neuroprotection by controlling many metabolic processes, including the release of mitochondrial cytochrome C, which inhibits the generation of reactive nitrogen species (RNS) and ROS. MNPs prevent the activation of macrophages and microglia in brain cells [35, 137]. AD symptoms are made worse by tau protein hyperphosphorylation and Aβ accumulation. Therefore, the main mechanism-based approaches of MNPs for neuroprotective action are reduction of Aβ plaque development, suppression of intracellular Aβ40 fibrillation, decomposition of Aβ fibrils into nontoxic fibrils, inhibition of tau hyper-phosphorylation, and Zn^2+^-Aβ mediated generation of ROS [35, 138]. The MNPs down-regulate NF-κB and IκB enzymes and decrease the levels of pro-inflammatory cytokines such as TNF-*α*, IL-1*β*, and IL-6 [35, 139]. By lowering caspase enzyme levels and preventing apoptosis, MNPs can regulate neuronal death. The symptoms of AD also decrease by a reduction in AChE enzyme levels [140]. MNPs contribute to neuroprotection by increasing the levels of phosphorylated-cAMP response element binding protein, hippocampal protein, and brain-derived neurotrophic factor [35, 140, 141].

#### Blood–brain barrier: different pathways and factors affecting the crossing of MNPs

For molecules and drugs, the blood–brain barrier (BBB) is a strong barrier [142]. Six distinct vital pathways for NPs, solute molecules, and drugs can traverse the BBB [143]. The first is “passive paracellular diffusion (PPD)”, which involves the transfer of materials between epithelial cells via intercellular spaces. This route is virtually nonexistent in a normal BBB because of the tight junctions. The second way is called “passive transcellular diffusion (PTD)”, in which molecules go past the bilayer cell membrane and into the intracellular space. Solute carrier proteins (SCP) are the third type of “carrier-mediated endocytosis”, in which solute molecules attach to membrane protein carriers, likewise ranging in concentration from high to low [143–145].

The fourth strategy is simple diffusion through a “receptor-mediated transcytosis (RMT),” in which a serum protein binds to its transcytosis receptor on the apical side to induce the development of the transcytosis vesicle; the second method, known as “adsorptive-mediated transcytosis (AMT),” engages proteins that are negatively charged on the surface of endothelial cells to transport drugs across the BBB. For the biggest molecules, such proteins, lipoproteins, or peptides, the transcellular pathway across the BBB endothelial cells necessitates their contact with particular receptors and transcytosis processes, according to certain research [144, 145].

This RMT is used by lipoproteins, insulin, lactoferrin, and transferrin. AMT and RMT are the main strategies used to transfer MNPs to the central nervous system [142, 144–147]. The last route is tight junction modulation (TJM), which means that very few molecules may diffuse across two adjacent BBB endothelial cells because TJs close the paracellular gap [148]. Thus, following concentration gradients, small lipophilic compounds (< 500 Da) may passively permeate over the BBB’s endothelial cells’ membranes [149]. Numerous physico-chemical factors, such as the molecular weight of the molecule, the lipophilicity, charge, size, number of bond donors and acceptors, and polar-surface area, all have a significant impact on this phenomena. The size of MNPs has a major impact on their ability to pass the BBB, which is essential for efficient transfer to the central nervous system. Of the various types of MNPs, AuNPs have a significance role for delivering drugs to the brain over the BBB to treat neurodegenerative illnesses. Smaller NPs are better at crossing the BBB, and research has indicated that permeability via BBB gaps increases as NP size decreases [150, 151]. AuNPs measuring 2.5 nm have been demonstrated by Sela et al., to migrate spontaneously over the BBB [152]. However, NPs smaller than 10 nm can readily pass through blood vessels and be eliminated by the kidneys [153]. In order to target drug delivery over the BBB, some studies employ NPs larger than 10 nm in diameter. In contrast to 3 nm and 120 nm particles, Ohta et al., demonstrated that 15 nm AuNPs had a better transport efficiency into the mouse brain [154]. According to some studies, the ideal sizes range from 50 to 100 nm. As reported by Hansen and Nichols [155] and McMahon and Boucrot [156], these diameters fall within the same range as the clathrin-coated vesicles and caveolae, which are 70–200 and 50–100 nm, respectively. According to several researches, the size of NPs has an inverse relationship with their passage [157]. When compared with 50 nm and 70 nm AuNPs, the researchers found that the 20 nm AuNPs accumulated the most in the brain. The ideal size for efficient delivery can vary depending on the particular application and technique utilized to assist BBB crossing, even though smaller NPs often exhibit superior permeability [158].

The ideal size of NP should be established according to the kind of treatment system on the basis of NP, since it depends on the type of NP, related coating and surface proteins, and physiological performance of the BBB [145, 159].

Nonetheless, some researchers report that NPs larger than 500 nm can effectively pass the BBB, particularly when modified and/or PEGylated [160]. Electrostatic interactions involved in the interactions of NPs with the BBB in addition to the size affect the permeability. Due to the negative charge of the endothelial cells’ outer membrane, positively charged NPs have an easier time using the adsorptive transcytosis route than their neutral or negatively charged NPs. However, neutral or negatively charged NPs exhibit less protein adsorption, which results in prolonged circulation periods [145, 161]. Coating the NPs with substances like PEG, nevertheless, may also result in extended circulation times. The NPs are protected from opsonization and subsequent removal by the mononuclear phagocyte system by the steric barrier that PEGylation creates around them. The low cell absorption efficiency seen for PEG-coated NPs may be improved by using other zwitterionic compounds [145, 161, 162]. While neutral NPs and low quantities of anionic NPs had no effect on BBB integrity, a study of the rat brain by Lockman et al., showed that cationic NPs could have a toxic effect and damage the BBB [163]. On the other hand, in some studies, age has been shown to weaken the BBB. Furthermore, neuroimaging studies in ischemic stroke, amyotrophic lateral sclerosis, brain trauma, epilepsy, and AD show that the BBB may be compromised. This is also the case for systemic disorders such liver failure. Researchers used a block copolymer of poly (lactic-co-glycolic acid) (PLGA) and CD44-binding hyaluronic acid (HA) to create 200 nm-diameter particles in a recent study [164]. Researchers have found that when NPs bind to these CD44-expressing cells, they are kept in the hippocampus for a longer period of time rather than being rapidly washed out. Since the BBB is damaged, these NPs may exploit it to attach to reactive astrocytes and microglia cells in the hippocampal regions of AD patients. The findings of this investigation demonstrate that no NPs were detectedin the brains of young, healthy mice, indicating that their BBB were unbroken. Surprisingly, though, NPs were found in considerable quantities in the brains of normal aged mice, indicating that the BBB weakens significantly with age, even in AD-free mice. The elderly mice used in this study are only around 60 years old, which is the human age equivalent. Furthermore, they discovered that AD mice's hippocampi contained high levels of NPs at all ages, although the concentration in old AD mice was higher than that of young rats. This demonstrates that the NPs’ penetration over the BBB to deep brain regions impacted by AD is age- and disease-dependent [164]. In addition, several animal studies have provided direct evidence that NPs could reach and accumulate in the brain parenchyma, including the striatumand hippocampus. In the case of Au, transferrin-containing AuNPs can enter and accumulate in the brain parenchyma after a systemic administration in mice through an RMT pathway [165]. In the AD mice model, D-glutathionestabilized AuNPs can cross the BBB following intravenous administration, depict great inhibitory efficiency against Aβ42 aggregation, and do not pose neurotoxicity [166].

According to the things mentioned above, there are different pathways for NPs such as MNPs to cross the BBB, which vary depending on their physicochemical properties. Among them, age is an influential and important factor in the weakening of the BBB.

### The role of plants bioactive compounds in the treatment of AD

**Fig. 4:**
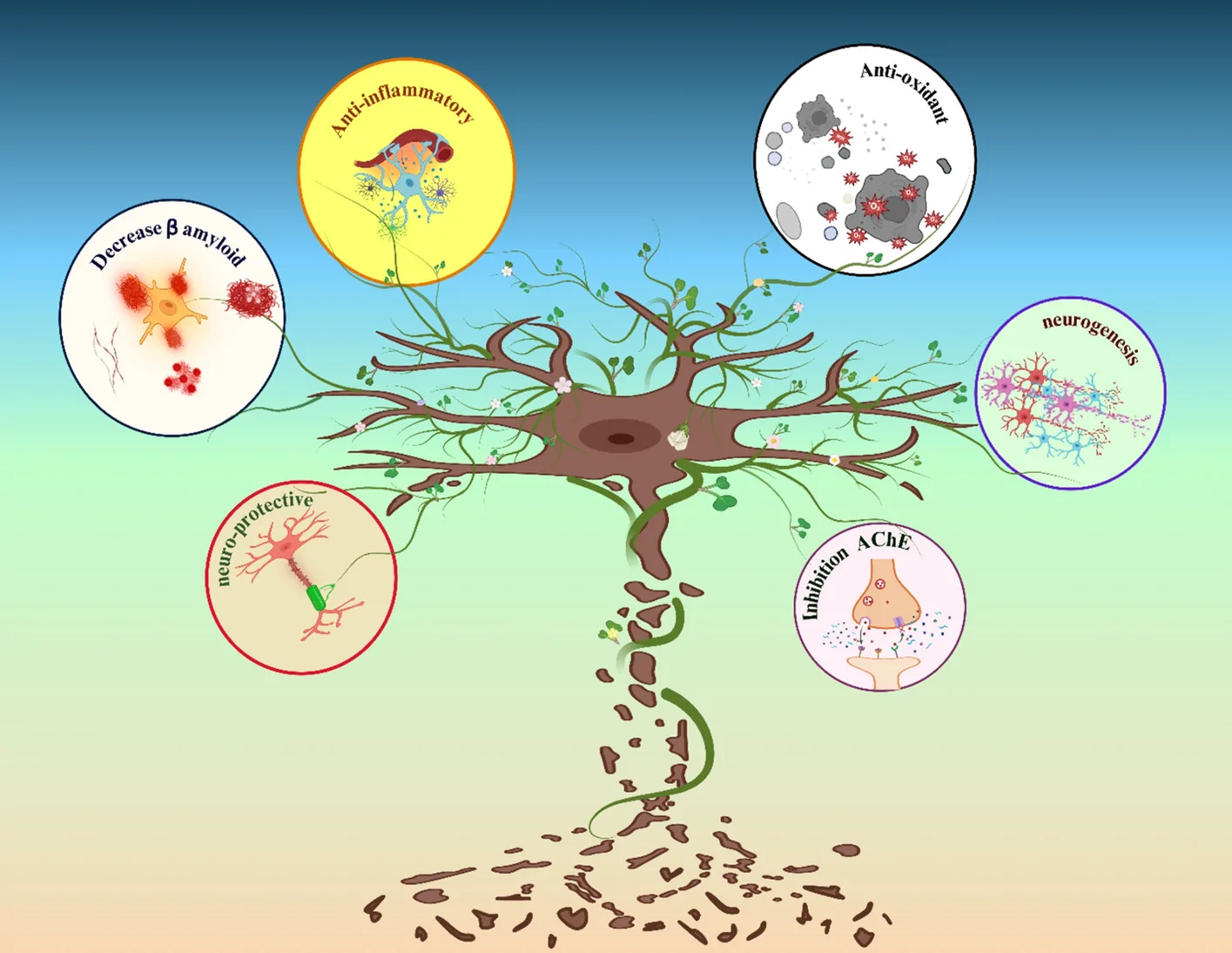
Different mechanisms of plant metabolites in reducing the effects of Alzheimer's disease. Created in BioRender. Jadidi Kouhbanani, M. A. (2026) https://BioRender.com/l67n859

Medicinal plants are growing in popularity as people’s concerns about the negative effects of synthetic pharmaceuticals grow. Because plant extracts can impact multiple targets simultaneously, they may be a more effective and innovative treatment option for AD than single drugs. Numerous taxa are included in a single group of anti-AD plants due to factors such as antioxidant potential, acetylcholine esterase inhibition, mitochondrial energy restoration, neuroprotection, and/or accelerated clearance of proteins [167, 168]. Plants' potential for therapeutic use based on their anti-inflammatory, antioxidant, neuroprotective, and anti-AChE characteristics is illustrated in Fig. 4.

These medicinal plants’ secondary metabolites may be used to treat AD and other common pathological brain diseases. Among the vital secondary metabolites of plants that are important in preventing AD neurodegeneration are phenolic acids, flavonoids, and alkaloids [167–170]. The secondary metabolites of these plants are crucial for preventing neurodegeneration or promoting regeneration. Flavonoids and other dietary bioactive phytochemicals have been shown in multiple studies to impact the pathophysiological processes underlying AD. Flavonoids are an effective addition to manufactured treatments for neurodegenerative diseases (NDDs) [171–173]. These phytochemicals are biologically active and have promising pharmacological properties, such as antioxidant, antiviral, antiallergic, antitumor, anti-inflammatory, antiplatelet, and antiapoptotic effects. Flavonoids slow the progression of AD by downregulating the production of oxidative stress (OS), tumor necrosis factor (TNF-α), and nitric oxide (NO) [173].

Furthermore, traditional medicinal plants like *Panax ginseng*, *Huperzia serrata*, *Ginkgo biloba*, and *Curcuma longa (turmeric)* may be utilized as a therapeutic option for the treatment of AD. Terpenoids and flavonoids found in *Ginkgo Biloba* have neuroprotective properties by improving cerebral blood flow and decreasing Aβ deposition. Curcumin has antioxidant and anti-inflammatory properties that may help reduce oxidative stress and neuroinflammation. A natural source of *Huperzine A*, *Huperzia Serrata*, improves cholinergic function by inhibiting acetylcholinesterase. *Ginsenosides* from *Panax Ginseng* have demonstrated anti-amyloidogenic and neuroprotective qualities [174, 175].

**Fig. 5:**
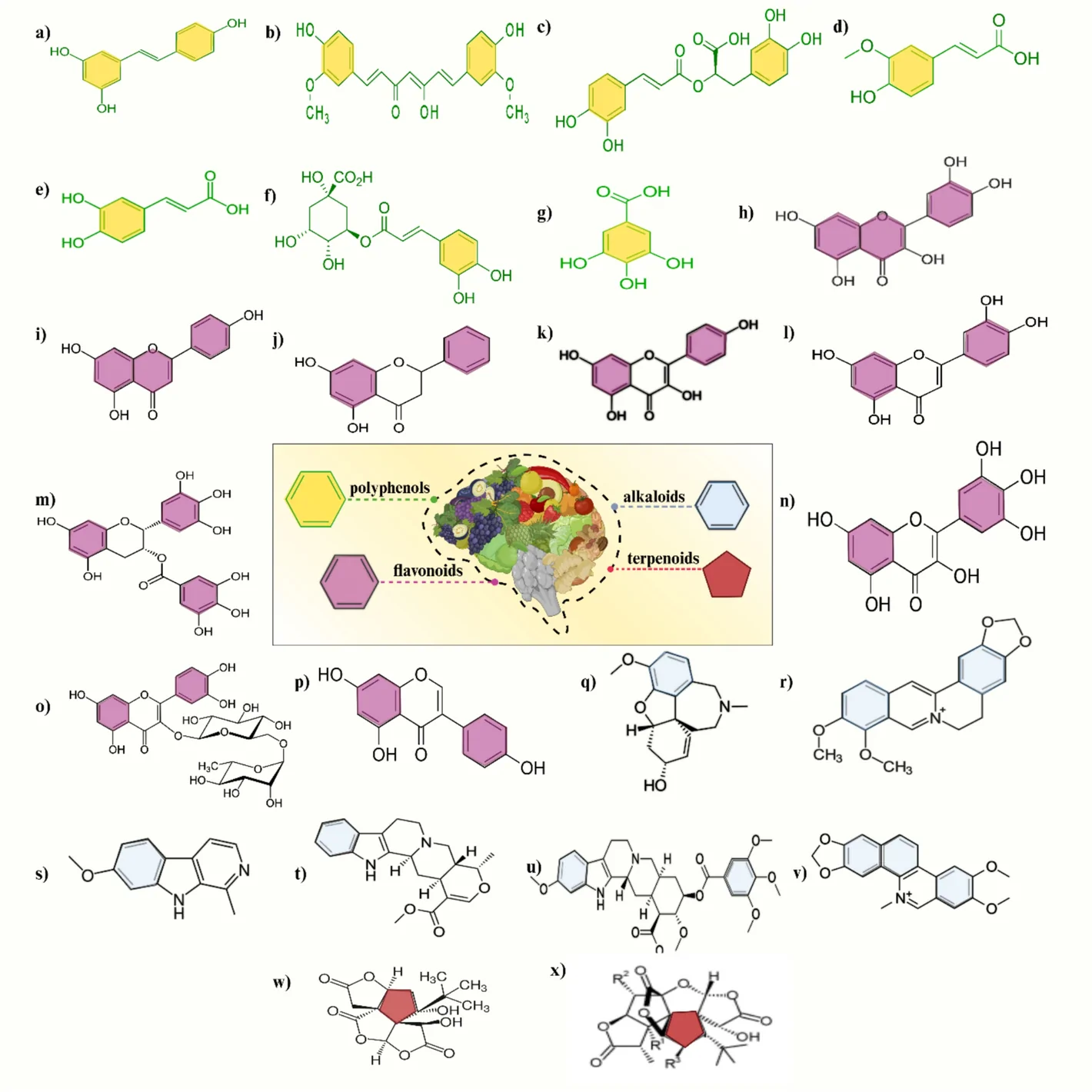
The structure of natural products with their classes. **a **Resveratrol, **b** Curcumin, **c** Rosmarinic acid, **d** Ferulic Acid, **e** Caffeic Acid, **f** Chlorogenic Acid, **g** Gallic acid, **h** Quercetin, **i** Apigenin, **j** Pinocembrin, **k** Kaempferol, **l** Luteolin, **m** Epigallocatechin-3-O-gallate, **n** Myricetin, **o** Rutin, **p** Genistein, **q** Galantamine, **r** Berberine, **s** Harmine, **t** Ajmalicine, **u** Reserpine, **v** Nitidine, **w** Bilobalide, **x** Ginkgolides. Created in BioRender. Jadidi Kouhbanani, M. A. (2026) https://BioRender.com/h4ji6jy

Many studies on AChE inhibitors have also been published in recent years. Numerous plants with promising AChE inhibitory activity have long been used as medicine in a variety of traditional practices [176]. AChE inhibitory activity has been found in a vast array of chemically diverse substances, such as terpenoids, alkaloids, flavonoids, and other phenolic compounds [132, 177]. For instance, among various plant extracts, the dry plant parts from *Terminalia chebula* (fruit), *Rauvolfia serpentine* (root), *Emblica officinalis* (fruits), *Nelumbo nucifera* (flower), and *Nardostachys jatamansi* (rhizome) were found to be the most effective sources of AChE inhibitors [178, 179]. Additionally, these inhibitors may be found in the plant families of *Valerianaceae*, *Myristicaceae*, *Amaranthaceae*, *Rutaceae*, *Amaryllidaceae*, and *Lycopodiaceae* [180, 181]. For this reason, the focus on treating AD has remained on plant-based AChE inhibition. On the other hand, essential oils derived from plants such as *Lavandula spp.*, *Persicaria hydropiper (L.), Delarbre (syn. Polygonum hydropiper L.)*, *Hedychium gardnerianum Sheppard ex Ker Gawl*, *Zosima absinthifolia Link*, and *Coriandrum sativum L.* have been used to treat AD [182, 183]. In addition to the plants mentioned above, Table 2 lists a large number of plants that have been effectively used to treat AD. Figure 5 shows the structures of the natural products under study along with their classification.

**Table 2:** Medicinal plants to anti-Alzheimer’s activity

No	Plant extracts			Anti-Alzheimer disease activities	Year	Ref
	Plants	Plant tissues for extraction	Bioactive phytocompounds	Properties		
1	*Fritillaria cirrhosa D.Don*	Fruit	Flavonoids & Phenolic	AChE inhibitor	2025	[184]
2	*Uncaria*	–	Alkaloids	AChE inhibitor	2025	[185]
3	*Jaboticaba*	Peel	*Flavonoids*	AChE inhibitor	2024	[186]
4	*Mazus pumilus*	*Whole plant*	*Alkaloids & Flavonoids*	*Inhibition of α-amylase & AChE*	*2024*	[187]
5	*Whit Willow*	*Bark*	*Phenolics & Flavonoids*	*Inhibitors of AChE & BChE*	*2024*	[188]
6	*Diplazium esculentum*	*Leaves*	*Alkaloids, Flavonoids, Glycosides & Phenolic*	*AChE inhibitor*	*2024*	[189]
7	*Psidium guajava*	*Fruit*	*Phenolic acids, Flavonoid & Alkaloids*	*AChE inhibitor*	*2024*	[190]
8	*Ginkgo biloba*	leaves	Ginkgolide & Bilobalide	AChE inhibitor	2024	[191]
9	*Crinum asiaticum var. sinicum*	Whole plant	*Alkaloids*	AChE inhibitor	2024	[192]
10	*Atriplex halimus L*	Saltbush	Phenols & Flavonoid	AChE inhibitor	2024	[193]
11	*Orthosiphon aristatus*	Whole plant	Phenols & Flavonoid	β-amyloid inhibitory	2024	[194]
12	*Spondias mombin, Carica papaya and Kalanchoe crenata*	Leaves	Flavonoids, quercetin & kaempferol	AChE inhibitor	2024	[195]
13	*Hypericum heterophyllum*	Leaves	Flavonoids	AChE inhibitor	2024	[196]
14	*Withania somnifera*	-	Alkaloids	Inhibition of Aβ	2024	[197]
15	*Punica granatum*	Peel	Polyphenols	inhibitors of AChE & BChE	2023	[198]
16	*Abelmoschus eculentus*	Seed	*Phenol* & *Flavonoids*	AChE inhibitor	2023	[199]
17	*Ceratonia siliqua L*	leaves	*Phenolic* acids, gallotannins & *Flavonoids*	AChE inhibiting effects in amyloid-β42	2023	[200]
18	*Camellia sinensis*	leaves	catechins, *Flavonoids*, anthocyanins & *Phenolic* acids	Inhibitors of AChE and BChE	2023	[201]
19	*laurel*	Leaves	*Flavonoids*	AChE inhibitor	2023	[202]
20	*Dolichandrone serrulata*	seem	*Alkaloids*, *Flavonoids*, & *Phenolic*	AChE inhibitor	2023	[203]
21	*Cassia spectabilis*	Flower	*Flavonoids*	Inhibitors of AChE and BChE	2023	[204]
22	*Macleaya cordata*	aerial part & Roots	Alkaloids	Inhibitors of AChE & BChE	2022	[205]
23	*Myristica fragrans*	Powder of plant	Flavonoids	AChE inhibitor	2022	[206]
24	*Panax ginseng*	Root	Ginsenosides	AChE inhibitor	2021	[207]
25	*Ashwagandha*	Root	Phenolic	Improving Memory	2021	[208]
26	*Curcuma longa*	Rhizome	Phenols, Flavonoids & Curcumins	Neuroprotective	2020	[209]
27	*Tinospora cordifolia*	Leaves	Flavonoids	AChE inhibitor	2020	[210]
28	*Ferula asafetida*	Whole plant	Coumarins & Other terpenoids	Regulation of Aβ plaques and hyperphosphorylated tau proteins	2020	[211]
29	*Melissa officinalis*	Leaves	Flavonoids	Aβ & AChE inhibitor	2019	[212]
30	*Myristica fragrans*	Leaves	Flavonoids	AChE inhibitor	2019	[213]
31	*Magnolia officinalis*	Stem bark	Phenolics & Flavonoids	AChE inhibitor	2019	[214]
32	*Rosmarinus officinalis*	Whole plant	Rosmarinic acid	Inhibition of Aβ accumulation	2018	[215]
33	*Rhodiola rosea*	Leaves & Root	Terpenoids, Alkaloids & Phenols	Improving Memory	2018	[216]
34	*Elatostema papillosum*	Leaves	*Phenolic* and *Flavonoid*	Inhibition of AChE & BChE	2018	[217]
35	*Irvingia gabonensis*	Bark	*Phenolic*	Inhibition of AChE & BChE	2018	[218]
36	*Withania somnifera*	Standardised extract	Alkaloids	AChE inhibitor	2017	[219]
37	*Ashwagandha*	Root	Phenolic	AChE inhibitor	2017	[220]
38	*Ashwagandha*	Root	Phenolic	Improving Memory and Cognitive Functions	2017	[221]
39	*Ginger*	Root	*Alkaloids*, *phenolics*,. *Flavonoids* & *Terpenoid*	AChE inhibitor	2017	[222]
40	*Albizia adianthifolia*	Leaves	*Alkaloids*, *Phenolics*, *Flavonoids* & *Terpenoid*	AChE inhibitor	2017	[223]

#### The role of polyphenol in AD

*Resveratrol:* The polyphenolic phytoalexin resveratrol (C_14_H_12_O_3_) is found in berries, grapes, and red wine. Resveratrol promotes the breakdown of the amyloid precursor protein in a non-amyloidogenic manner. It also improves the removal of Aβ peptide and reduces damage to neurons. Most of the recent experimental research on resveratrol and AD has been conducted both in vitro and in vivo using a variety of experimental models [224, 225]. The use of polyphenols derived from grape seeds reduced the oligomerization of Aβ peptide and improved the cognitive impairment in Tg2576 mice, according to Wang et al. [226]. It has been shown that resveratrol inhibits the production of amyloid and significantly reduces the number of activated microglia in APP/PS1 mice [225, 227]. Resveratrol stopped processing the amyloid precursor protein in both strains in an amyloidogenic way by high-fat diets (HFD) and restored the ubiquitin–proteasome system’s exceptionally high proteolytic activity, indicating that a compensatory mechanism is in place to stop the accumulation of abnormal proteins [225, 228]. Additionally, dietary resveratrol (1 g/kg in the diet/7 months) has a significant neuroprotective effect, increased life expectancy, decreased cognitive impairment, and decreased tau hyperphosphorylation and amyloid load in the in vivo experiment using SAMP8. They also found that the non-amyloidogenic ADAM-10 enzyme and the AMPK and pathways are activated upon resveratrol supplementation [109, 229].

*Curcumin:* The main source of curcumin (C_21_H_20_O_6_), a food preservative, taste enhancer, and spice is the rhizome of turmeric (Curcuma longa). One interesting finding is that the incidence of AD in people aged 70–79 is four times lower in India than in the US, suggesting that older Indians’ lower risk of AD may be due to a diet rich in curcumin [225, 230]. This drug is helpful in AD because in addition to its anti-tau action, it also has anti-secretase, anti-cholinesterase, anti-amyloidogenic, and down-regulates BACE1 expression properties [109]. Curcumin reduced the production of Aβ by inhibiting the activation of GSK-3β dependent PS-1 and thereby lowering the expression of the GSK-3β and PS-1 proteins, according to Zhang et al., [231]. Additional roles of curcumin include preventing neurotoxicity and removing tau tangles.

*Rosmarinic acid:* A naturally occurring phenolic compound with a wide range of biological activities is rosmarinic acid (RA). It has two hydroxyl groups in ortho-position on each of its two phenolic rings. A carboxylic acid group, a carbonyl group, and an unsaturated double bond bind the two rings together [232]. This phenolic compound is present in a number of medicinal plants, herbs, and spices including *Salvia officinalis, Ocimum basilicum, Heliotropium foertherianum, Rosmarinus officinalis, Perilla frutescens, Melissa officinalis,* and several other medicinal plants, spices, and herbs [232, 233]. It protects PC-12 cells from the harm that amyloid peptide causes. RA acts through methylation, dehydroxylation, and sulfate conjugation when taken orally [225]. As part of its neuroprotective effects, RA inhibits apoptosis by reducing the levels of ROS and caspase-3. It also inhibits the activation of p38 MAPK, which is a factor in neuronal degeneration [225, 234].

*Ferulic acid:* 3-methoxy-4-hydroxycinnamic acid, or ferulic acid (FA), is present in whole grapes, grains, spinach, parsley, cereal seeds, coffee, artichokes, and cereal seeds, especially those of rye, oats, wheat, and barley.[235].

FA is a key component of traditional Chinese medicine (TCM) that possesses anti-inflammatory and antioxidant properties while also preventing Aβ aggregation. According to reports, FA derivatives have excellent biological activity, minimal toxicity, and high BBB permeability [236]. According to earlier research, FA or its derivatives can improve AD cell model resistance and symptoms while also having a variety of therapeutic effects on AD models. FA and its derivatives are prospective medications for the multi-targeted therapy of AD because of their anti-Aβ aggregation, anti-inflammatory, and antioxidant properties. According to the findings of the Zhou et al., study, FA and its derivatives significantly improve AD in animal and cell models, indicating that they might be useful medications for AD patients [236].

*Caffeic Acid:* The chemical molecule known as caffeic acid (CA) has two functional groups: acrylic acid and phenolic hydroxyl. Numerous pharmacological characteristics, including anti-inflammatory, antibacterial, antiviral, anticancer, antioxidant, and neuroprotective qualities, are demonstrated by this substance, which is abundantly found in medicinal plants, vegetables, and fruits [237, 238]. A research demonstrated that, by administrating CA (30 mg/kg b.w./day) for 30 weeks, the memory and learning deficits in high-fat (HF)-induced hyperinsulinemic rats were dramatically ameliorated. Additionally, the hippocampal levels of *β*-amyloid 1–42 (Aβ1-42) were decreased in CA-treated hyperinsulinemic rats due to the attenuation of APP and *β*-site APP cleaving enzyme expression [238].

Chlorogenic acid: The phenolic acid known as chlorogenic acid (CGA), with the chemical formula C16H18O9, is created when CA and quinic acid, a phenylpropanoid compound generated by the shikimic acid pathway during aerobic respiration in plants, combine [209, 210]. Foods including apples, cocoa, berries, tea, coffee, and others contain CGA [239]. In the brain, CGA and its metabolites can be widely distributed and are permeable to the blood–brain barrier. CGA is linked to AD and possesses neuroprotective qualities. Aβ aggregation is inhibited by CGA, which stops the production of the Aβ1–42 *β*-sheet. The caffeoyl group, which may create covalent connections with Aβ1−42 residues to prevent the formation of Aβ oligomers and destabilize Aβ42 aggregates, mediates this characteristic [240]. Through the Wnt signaling pathway, CGA can prevent the cognitive impairment in early AD rats. Additionally, research has demonstrated that CGA can prevent NF-κB and its signaling pathway from being activated, which reduces neurotoxicity and delays the onset of AD [241].

Gallic acid: Gallic acid (GA), a phenolic acid that is often referred to as 3, 4, 5-trihydroxy benzoic acid, has been linked to a variety of neurological conditions. It is a secondary metabolite that may be found in free-state or ester form in a variety of locations across the higher plant kingdom [242]. GA’s antioxidant qualities and capacity to eliminate oxygen free radicals make it a promising treatment option for neurodegenerative diseases [243]. GA may be able to pass across the BBB and affect peripheral tissues as well as the central nervous system [244, 245]. By encouraging neurogenesis in the hippocampus dentate gyrus zone, GA enhanced spatial memory, as demonstrated by Ding et al. In APP/PS1 mice, it also enhanced synaptic plasticity, decreased tau phosphorylation and amyloid-*β* concentration, and raised synaptic protein levels. Moreover, GA suppressed GSK-3*β* (glycogen synthase kinase) activity [246].

#### The role of flavonoids in AD

By preventing Aβ and tau pathology, Aβ deposition, APP processing, neuroinflammation, oxidative stress, and neurogenesis, flavonoids like pinocembrin, apigenin, and quercetin (QT) help AD progress. Numerous studies suggest that the flavonoids may be promising treatments for neurological conditions [247].

*Quercetin:* QT (C_15_H_10_O_7_) is a flavonoid with significant pharmacological and medicinal properties. The protective effects of QT on the brain have been thoroughly investigated [225]. QT at low pretreatment doses significantly improved Aβ (1–42) induced protein oxidation, lipid peroxidation, apoptosis, and cytotoxicity in cultured neurons, according to the in vitro study by Ansari et al., [248]. However, at high doses, QT was toxic and did not provide neuroprotection. Furthermore, in the CA1 hippocampus region of aged triple transgenic mice with AD, QT was able to decrease Aβ plaque aggregation and pro-inflammatory mediators like COX-2, IL-1*β*, and iNOS [109].

Apigenin: Apigenin is a flavonoid that is widely present in vegetables such as belongs, cabbage, celery, and bell pepper. Apigenin has demonstrated protective effects against Aβ peptide (25–35)-induced toxicity in mice by improving cerebral blood flow, modulating the acetylcholinergic system, lowering oxidative assault, and improving cognitive functions and memory [249, 250]. Follow-up studies by Liu et al., show that Apigenin treatment of neuronal cells protects against amyloid-induced toxicity by regulating the genes that encode Cu homeostasis [251].

Pinocembrin: A naturally occurring flavonoid called Pinocembrin has been extracted from a variety of plants, mainly the heartwood of *Populus tremula*, *Pinus sylvestris*, *Sparattosperma leucanthum*, and *Eucalyptus globulus* [225]. *Propolis* also contains a lot of this flavonoid. In one study, Pinocembrin was used to improve memory and cognitive function in the AD mouse model while also reduced the neuronal degeneration in the cerebral cortex [252]. When taken orally, Pinocembrin was rapidly absorbed into the large intestine and had an hour-long half-life. When administered orally to mice, Pinocembrin has been shown to inhibit inflammatory pathways like NF-KB and MAPK. This leads to apoptosis in mice receiving cerebral infusions of Aβ peptide (25–35). When exposed to Aβ peptide (1–40), Pinocembrin was found to have a similar antitoxic effect on microvascular endothelial cells in the human brain. Additionally, it demonstrates neuroprotection by inhibiting the Nrf2/HO-1 pathway, which protects human neuroblastoma cells from Aβ-induced toxicity [225, 252–254].

*Kaempferol:* The flavonoid kaempferol (KMP) has many derivatives, such as kaempferol-O-rhamnoside, 3-O-β-rutinoside/6-hydroxykaempferol 3, 6-di-O-β-d-glucoside, and kaempferide [255]. KMP has neuroprotective properties against the onset of several neurological illnesses, including AD, and is present in a wide variety of plants, including beans, tea, strawberries, and broccoli. Because KMP can penetrate the BBB, it has a stronger protective impact [256]. By preventing the deposition of amyloid fibrils (like tau, Aβ, and *α*-synuclein), inhibiting microglia activation, reestablishing the mitochondrial membrane to prevent oxidative stress, lowering the release of inflammatory factors, shielding the BBB, and blocking certain enzyme activities (like cholinesterase), KMP and its derivatives primarily provide neuroprotection [257].

*Luteolin:* Fruits and vegetables including apple peels, celery, broccoli, parsley, carrots, peppers, cabbages, chrysanthemum flowers, and onion leaves are rich sources of luteolin (Lu), a flavonoid [240]. Following oral ingestion, Lu can be found in brain tissues and is BBB permeable. Most likely, an adenosine triphosphate-binding cassette transporter provides access to the brain [258, 259]. Treatment with 3–30 μM increased the coculture’s cell viability in a concentration-dependent manner in an in vitro BBB model exposed to Aβ1-40 fibrils. Additionally, the 10 and 30 μM treatments improved BBB permeability and preserved BBB function [240]. By activating the gut microbiota–liver–brain axis, Lu (10–70 mg/kg bw for rats) can affect Aβ deposition, decrease AD progression directly, and modify systemic and brain insulin resistance [259, 260].

Epigallocatechin-3-O-gallate: Many studies have revealed the potential neuroprotective effect of Epigallocatechin-3-O-gallate (EGCG), a major catechin derived from the polyphenolic portion of green tea, on animal models of AD [261]. By triggering signaling pathways linked to neuronal survival and controlling inflammatory processes implicated in neurodegeneration, such as ferroptosis and microglia-induced cytotoxicity, EGCG functions as an antioxidant [261]. EGCG decreased the formation of Aβ in Tg2576 AD mice and neurons overexpressing APP695 [262]. It also reduced plaque and Aβ levels. These results showed that EGCG increases the synthesis of the soluble amyloid precursor protein-C-terminal fragment of APP, which in turn activates the non-amyloidogenic α secretase proteolytic pathway [225, 262, 263].

*Myricetin:* vegetables, berries, Tea leaves, and medicinal herbs are just a few of the plants that contain myricetin, a naturally occurring flavonoid. There is increasing evidence that myricetin supplements have therapeutic effects for several illnesses of the neurological system, including AD [264–266]. In one study, myricetin's impact was evaluated in 3 × Tg mice and neuronal SH-SY5Y cells treated with Aβ42 oligomer. According to the findings, myricetin improved tau phosphorylation and decreased pre- and postsynaptic protein levels in 3 × Tg mice and neuronal SH-SY5Y cells treated with Aβ42 oligomer. Furthermore, myricetin repaired mitochondrial dysfunction through the related GSK3β and ERK 2 signaling pathways and decreased the production of reactive oxygen species, lipid peroxidation, and DNA damage [267].

Rutin: Under pathological circumstances, rutin, a multipurpose natural flavonoid glycoside, has a significant impact on the different cellular functions. It has also been demonstrated that rutin and/or its metabolites can alter the cognitive and behavioral symptoms of neurodegenerative illnesses since they can pass across the BBB [268]. In vitro, rutin has been demonstrated to reduce and reverse the development of Aβ25–35 fibrils, suggesting that its effect may be related to its capacity to scavenge free radicals and may mitigate neurotoxicity [269]. For instance, applying rutin-loaded NPs to human neuroblastoma SH-SY5Y cells resulted in reduced NO and ROS levels as well as protective benefits against Aβ-induced cytotoxicity [270]. According to Xu et al., rutin attenuated memory deficits in APPswe/PS1dE9 transgenic mice after oral administration of 100 mg/kg per day for six weeks. It also decreased oligomeric Aβ levels, downregulated microgliosis and astrocytosis, and decreased IL-1 and IL-6 levels in the brain [271].

*Genistein:* A phytoestrogen found in seeds, vegetables, and fruits which shows tremendous potential in preventing a range of damage caused by Aβ [272]. Because of its potential to improve Aβ-induced impairment and its antioxidant ability to scavenge the free radicals generated in AD, genistein has received a lot of attention. In addition, in order to prevent AD, it can directly interact with the specific signaling proteins and stabilize their activity [109, 273]. Zhou, X. et al., showed that in the in vitro model, genistein therapy increases cell survival and reduces inflammatory damage caused by A25–35 in BV-2 microglia cells through the Toll-like receptor 4 (TLR4)/NF-kB signal pathway [274]. Furthermore, by lowering Aβ (1–42)-induced cell death and genotoxicity in the SH-SY5Y cell line in vitro, it was discovered that galantamine and genistein combinations have strong neuroprotective activity [275]. Similar to this, a number of in vivo studies have shown that genistein, a selective estrogen receptor modulator (SERM), can improve brain function across the BBB, decrease Aβ protein aggregation-induced neurotoxicity, and have neuroprotective effects [108].

#### The role of alkaloids in AD

A naturally occurring class of organic nitrogen compounds, alkaloids are mostly found in plants especially in flowering plant families like the *Solanaceae (nightshades), Papaveraceae (poppies), Amaryllidaceae (amaryllis), and Ranunculaceae (buttercups)* [109, 276].

The Lycopodium alkaloids are pyridine, quinolizine, or *α*-pyridone alkaloids, which can be classified into four distinct classes: flabellidine or lycodine, lycopodine, miscellaneous, and fawcettimine. Huperzine A, the most significant member of this group, is an alkaloid that resembles lycodine. It was initially isolated from the Chinese lycopod *Huperzia serrata* and has been used for centuries to treat schizophrenia, fever, inflammation, swelling, and strains. In AD, it demonstrated neuroprotective action against neuronal apoptosis, which can be mediated through increased expression of antiapoptotic proteins/genes and the regulation of pro-apoptotic protein expression [109, 276, 277].

*Galantamine*: The first nutraceutical to receive an FDA license in 2001 for reversible AChE inhibitory activity was GAL. It is an alkaloid in the Amaryllidaceae family and sold by Janssen under the brand name Reminy [278]. The US, Czechia, Thailand, Switzerland, South Africa, Mexico, the European Union (apart from the Netherlands), Poland, Singapore, Korea, Iceland, Norway, Canada, Argentina, and Australia are among the 29 countries where GAL has been approved by regulators. Although it is currently synthesized, it can be derived from *Galanthus nivalis*, *Lycoris radiate*, *Narcis sus tazetta*, and *Leucojum atrium*. Interest in plant therapies for AD has increased as a result [109, 278, 279].

By increasing acetylcholine levels in the brain, the acetylcholinesterase inhibitor GAL helps treat AD by enhancing memory and cognitive performance [280, 281]. Compared to BuChE, it is 10–50 times more selective for acetyl. Competitive inhibitors should theoretically have a greater impact in regions with low levels of ACh and less impact in regions where acetylcholine levels are higher. This could have a selective impact on the parts of the brain with lower ACh levels that are impacted by AD. ACh levels in the synaptic cleft rise when GAL binds to brain AChE and decreases ACh catabolism [282, 283].

The goal of numerous studies has been to clarify GAL’s neuroprotective benefits. Additionally, recent cellular research has demonstrated that GAL can shield SH-SY5Y cell lines from toxicity caused by Aβ. Further in-depth research to comprehend the molecular and chemical characteristics linked to GAL-protective properties has been validated and supported by these investigations. Furthermore, it potentiates cholinergic nicotinic neurotransmission by acting as an allosteric modulator at nicotinic cholinergic receptor sites [109, 119].

Additionally, GAL administration at 8–32 mg/day consistently improves cognitive functions and activities of daily living in patients with mild to moderate AD over a period of 3–6 months, according to clinical studies on the drug. Additionally, the effects of GAL (24 mg/day) last for a sustained year. For mild to moderate AD, GAL is now prescribed in sustained-release capsule form [284, 285].

Furthermore, over the course of 6 months, a different study demonstrated that GAL can stabilize and improve behavioral symptoms, cognitive function, and activities of daily living. Its effectiveness and tolerability are comparable to those of other ChE inhibitors, such as donepezil and rivastigmine [281]. Recent research focuses on how chemicals work in concert. GAL and memantine combination has demonstrated dual-path neuroprotection by blocking AChE and reducing N-methyl-D-aspartate-induced neurotoxicity [280].

Berberine: *Goldenseal*, *Tree turmeric*, *Phellodendron*, *European barberry*, *Goldthread*, and *Oregon* grape contain the chemical Berberine (BB). The sedative effects of BB on the nervous system were initially reported in the 1970s. Anti-neuroinflammation, cholinergic system enhancement, and endoplasmic reticulum stress inhibition are just a few of the potential multi-targeted therapeutic effects of BB for the treatment of AD [225, 286, 287].

In neurons expressing the human Swedish mutant APP, BB prevents the production of pathogenic Aβ and Aβ aggregation. In APP/PS1 mice, BB ameliorates endoplasmic reticulum stress and consequently cognitive deficits [288]. In an animal model of AD, a study found that BB decreased APP expression, indicating that BB can process APP to lower Aβ. Aβ may be regulated by altering the hyperphosphorylation of Tau protein, BACE1, or the terminal fragment of APP (C99). By inhibiting the hyperphosphorylation of Tau protein and the synthesis of Aβ, BB has been demonstrated to slow the progression of AD. By activating the extracellular kinase 1/2 signaling pathway and inhibiting beta-secretase expression, BB lowers the production of Aβ40/42 [225, 289, 290]. ACh levels in the brain rise when ChE, the primary enzyme that breaks down ACh, is inhibited. ChE inhibitors have been the focus of a great deal of anti-AD pharmacological research in an effort to treat the cognitive symptoms of AD. Several tests have been conducted to ascertain the effect of BB on ChE activity. In rats treated with ethanol, BB reduced ChE activity and oxidative stress. After a month of BB treatment, rats’ memory impairment caused by streptozotocin treatment improved [225, 290, 291].

*Harmine and Harmaline:* The primary constituents of Peganum harmala L. (Nitraceae) are the β-carboline alkaloids harmine (HAR) and harmaline (HAL) [292]. According to studies, these alkaloids have MAO-A inhibitory, antioxidant, anti-inflammatory, and AChEI potential. They may also be able to penetrate the BBB. By limiting the production of maleic dialdehyde, inhibiting AChE, boosting the activity of glutathione peroxidase and superoxide dismutase, and decreasing inflammation by inhibiting nitrous oxide, TNF*α*, and myeloperoxidases, they improve cholinergic function through a variety of mechanisms [292–294]. HAL has been shown in studies to improve memory impairment in APP/PS1 transgenic AD mice and scopolamine-induced mice. This is likely due to HAL’s sequential BBB penetration, activation of certain early response genes, and acetylcholinesterase inhibition [293]. Furthermore, HAR may be effective in treating neurodegenerative diseases, as evidenced by a research that found oral administration of 20 mg/kg HAR improves memory impairment by enhancing cholinergic function in scopolamine-induced mice [294].

*Ajmalicine and Reserpine:* Indole alkaloids, which include substances like reserpine (RE) and ajmalicine (AJ), are a significant class of alkaloids that are produced from tryptophan and secologanin [280]. In Indian traditional medicine, AJ and RE have been used for ages to treat a broad range of illnesses, such as snake, malaria, fever, and bug stings, diarrhea, and stomachaches. Febrifuge, Insanity cure, and uterine stimulant were some of its other uses [292]. Rauwolfia serpentina Benth is the source of the indole alkaloids AJ and RE, which have the potential to be AChEI. Additionally, Aβ42 fibril production and other AD targets including BACE-1 and MAO-B were reduced by both AJ and RE [295, 296]. Furthermore, in the AD model of Caenorhabditis elegans, RE has been investigated for its capacity to protect against Aβ toxicity, which is characterized by paralysis [297]. This model aids in comprehending the potential mechanism of AD disease by expressing the human toxic Aβ1–42 in the muscle [298]. By postponing harmful Aβ expression-mediated paralysis, RE was able to mitigate the AD pathogenesis in C. elegans [297].

*Avicine, Nitidine, and Chelerythrine:* The alkaloids nitidine, avicine, and chelerythrine are all benzophenanthridines. In avicine, positions 8 and 9 are connected by a dioxomethylene bridge, but in nitidine, two methoxy groups take their place [292, 299]. Two alkaloids, ntidine and avicine, having dual cholinesterase inhibitory action against AChE and BuChE were extracted from Zanthoxylum rigidum Humb. Et Bonpl. Ex Willd. As mixed-type inhibitors, they have the ability to bind to the enzymes' catalytic and peripheral anionic sites (PAS) and prevent ChE-induced Aβ1–40 aggregation [292, 299]. The structural motif of nitidine is also present in chelerythrine, another benzophenanthridine alkaloid that is frequently isolated from Chelidonium majus L. (Papaveraceae). The location of one methoxy group on aromatic ring A is the only difference between the two alkaloids [300]. At 5, 10, and 100 µM, chelerythrine suppressed AChE-induced Aβ aggregation with 48.5%, 65.0%, and 88.4%, respectively. This suggests that chelerythrine is a strong inhibitor of AChE-induced Aβ1–40 aggregation [301].

#### The role of terpenoid in AD

Researchers have become interested in Terpenoids’ anti-ChE activity among their many biological activities. Accordingly, *bilobalide* and *ginkgolides*, two significant bioactive components of *Ginkgo biloba* extract (GbE), have been shown to exhibit neuroprotective effects in AD through a variety of mechanisms, including antioxidative and anti-inflammatory properties [302–304].

*Bilobalide*: *Ginkgo Biloba* preparations are the most common source of *bilobalide*, a naturally occurring sesqui-terpenoid. *Bilobalide* may have neuroprotective effects, according to evidence that has accumulated over the past few years. *Bilobalide* exhibits neuroprotective activity against amyloid damage by up-regulating the PI3-K pathway, which lowers the amount of *β*-APP and expresses soluble APP [225, 305]. In order to counteract amyloid toxicity, it also promotes activation of the BDNF and CREB pathways and protects neurons from synaptic loss. In hippocampus primary cultures, it exhibits neuroprotection. According to a recent study, it may also have antioxidant properties that help lessen the toxicity that amyloid causes. Another study has shown how it reduces toxicity by preventing the MAPK and NF-KB pathways from being activated. Additionally, it might stop Aβ fibrils from forming [225, 306, 307].

*Ginkgolides: Ginkgo biloba* contains Terpenic lactones called *Ginkgolides*, which are biologically active. The neuroprotective effects of EGb761, an extract of *G. biloba* leaves that contains 5–10% organic acids, 6% terpenoids, and 24% flavonoid glycosides, have been studied. The main pharmacologically active components of EGb761 are terpene trilactones *ginkgolides* [304, 308]. The functions and fundamental processes of *ginkgolide* (Baiyu^®^) were examined in transgenic mice with PREN 1/APP and BV-2, a murine microglial cell line. *Ginkgolide*-treated mice showed reduced escape latency, neuron loss, less inflammatory cell infiltration, and less plaque in the hippocampus compared to PREN 1/APP mice. Following *Ginkgolide* treatment, Aβ1–42-treated BV-2 cells or the brain tissue of PREN 1/APP mice also showed decreased levels of ASC, IL-18, IL-1β, NLRP3, ROS, and caspase-1. By inactivating the caspase-1/NLRP3 pathway, *Ginkgolide* protected AD patients, at least in part [304].

### Green synthesis: combiningz the potential of plant metabolites and metal nanoparticles

Chemical compounds found in medicinal plants can be used as soothing agents to treat AD [109]. Flavonoids and other plant metabolites, for instance, have antioxidant qualities that can lessen the oxidative stress linked to AD [309]. According to several studies, plants extract have shown promise in treating a variety of pathological conditions, including AD. For example, over 160 polyphenols, such as different flavonoids and phenolic acids, are found in the salvia plant, which is used to treat AD [170].

Certain plant extracts, such as essential oils, contain a class of compounds called terpenes which are capable of inhibiting AChE. The activity of various plant species) including *Malva silvestris, Melissa officinalis, Hypericum undulatum, Sanguisorba minor, Paronychia argentea, Mentha suaveolens, Laurus nobilis, Lavandula pedunculata, Lavandula angustifolia,* and *Salvia officinalis*) from interior Portugal, was assessed using decoction, essential oil, and ethanol extract. Among these, the plant extracts from *Mentha suaveolens* and *Melissa officinalis* demonstrated an AChE capacity inhibition activity greater than 50% in the essential oil fraction. A high Inhibition value of AChE in the ethanolic fraction was 68%, 64%, and 78% by extracts *Hypericum undulatum, Laurus nobilis,* and *Sanguisorba minor*, respectively. Higher values of AChE inhibitory activity using decoctions 82%, 69%, and 68% were demonstrated by extracts of *Hypericum undulatum, Mentha suaveolens* and *Lavandula pedunculata*, respectively [310].

On the other hand, the use of MNPs in nanomedicine-based methods for treating AD has been seen as a promising research area. For instance, in a rat model of sporadic AD-type dementia, MNPs, like AuNPs, inhibit inflammation, oxidative stress, and cognitive deficits [311]. Additionally, IONPs can be utilized to detect Aβ in order to help in the diagnosis and treatment of AD [312].

However, the synthesis of NPs using physicochemical methods has a number of drawbacks (cost and toxicity) that prevent them from being used as a potential nanomedicine [313]. It is important to remember that the chemical substances used in the chemical synthesis method stay attached to the surface of NPs, potentially making them toxic. Therefore, the environmentally friendly NPs synthesis is a new technique that uses pure phytochemicals or medicinal plants to stabilize and chelate biocompatible NPs. In recent years, a number of scalable, reproducible, simple, and easy green synthesis methods for NPs have been created. Because of this, a number of biological systems including bacteria, fungi, yeast, and plants extract are currently widely used in green synthesis techniques to produce MNPs [12, 314]. Because of the variety of plants and its ease of use, plant-based MNP green synthesis is currently considered a gold standard among these green biological techniques [109].

**Fig. 6:**
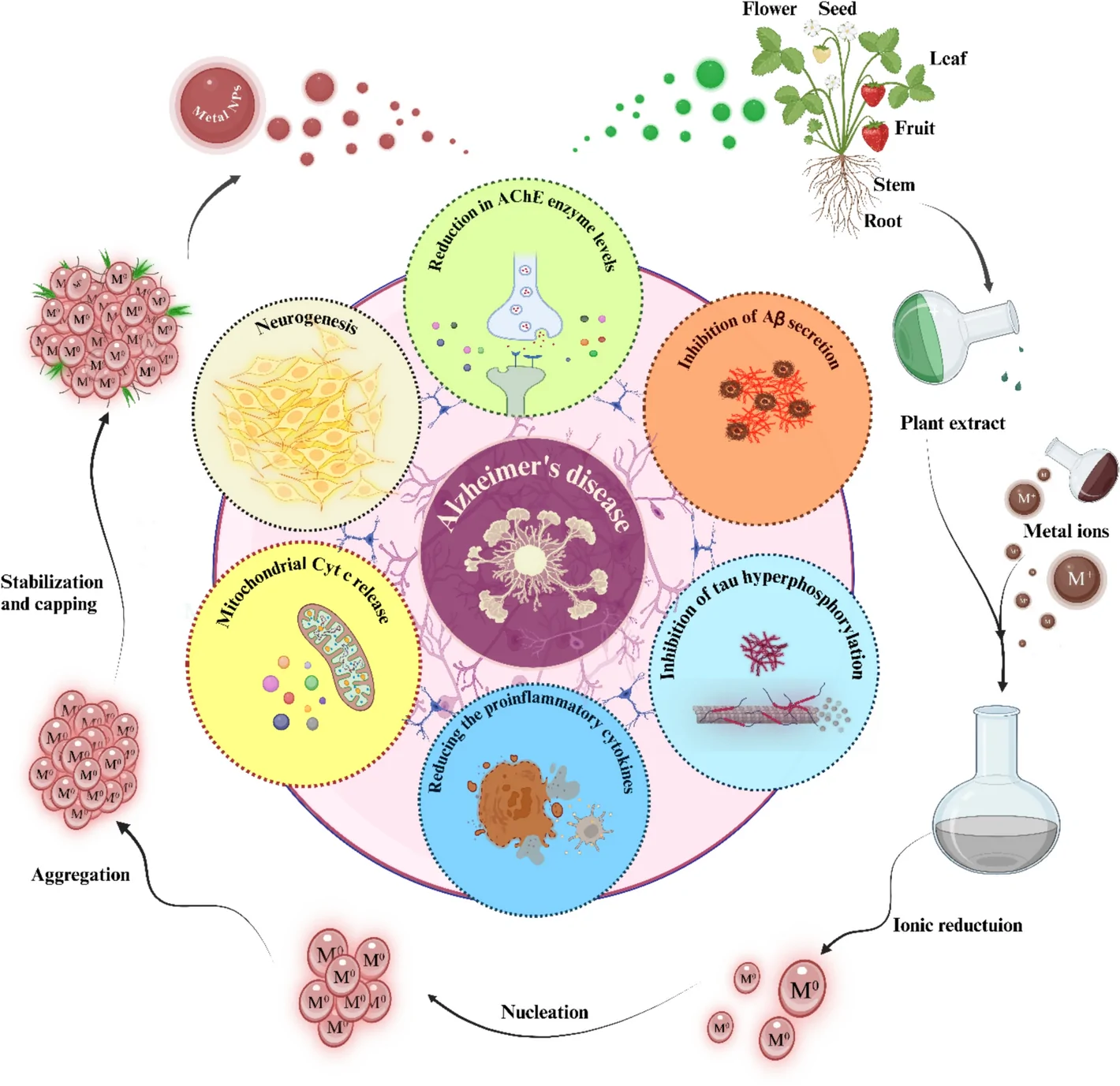
Combining the potential of plant metabolites and metal nanoparticles by green synthesis. Created in BioRender. Jadidi Kouhbanani, M. A. (2026) https://BioRender.com/m46q752

During the green synthesis of MNPs (viz. ZnONPs, AuNPs, and AgNPs) using plant extracts from *Sabal palmetto*, *Terminalia arjuna*, *Malephora lutea Schwantes,* and *Lampranthus coccineus,* it was shown that activities depicting phytochemicals as bioactive substances that are able to inhibit and slow down specific molecular cascades are linked to the development of AD. For the green synthesis of MNPs, biomolecules including phytochemicals (saponins, alkaloids, terpenoids, flavonoids, and polyphenols) serve as stabilizing, capping, and reducing agents [225]. Figure 6 shows the combining the potential of plant metabolites and MNPs by green synthesis.

Numerous studies have shown that the use of nanotechnology in plants extracts increases their effectiveness. Plant metabolites exhibit antioxidant properties and can be integrated with MNPs to improve their therapeutic efficacy against neurodegeneration. This approach leverages the unique properties of MNPs combined with the therapeutic potential of plant-derived compounds [109, 166]. In the following, the synergistic effect of MNPs and plant metabolites for the treatment of AD is discussed in detail.

A few studies have compared the AChE inhibitory activities of green-synthesised MNPs with those of standard drugs. For instance, in a study, the green synthesis of ZnO NPs was carried out using methanolic extracts of various plant parts of *Sabal blackburniana*, including pollen grains, fruits, and leaves. Tiny spherical particles with sizes ranging from 2.23 to 49.56 nm were revealed by TEM analysis as the most common morphology. The results showed that, compared with donepezil (IC_50_ = 50.7 ± 5.769 ng/mL) as the standard, the synthesized ZnO NPs exhibited strong AChE inhibitory activity, with IC₅₀ values of 117.95 ± 6.858, 81.985 ± 3.075, and 63.78 ± 1.0465 ng/mL for leaf, fruit, and pollen grain extracts, respectively [315]. According to another study, AgNPs synthesized using aqueous extracts of *M. lutea* and *L. coccineus* (with spherical morphology and size ranges of 18.66–28.19 nm and 12.86–16.31 nm, respectively) were assessed for their in vivo anti-AChE activity in mouse models. The AgNPs derived from *M. lutea* and *L. coccineus* demonstrated high anti-AChE activity at concentrations of 1.36 ng/mL and 0.82 ng/mL, respectively, with activity levels comparable to rivastigmine, a commonly used standard drug for neurodegenerative disorders. Furthermore, the authors proposed that these AgNPs are capable of crossing the BBB, leading to increased AChE regulation and reduced oxidative stress, thereby highlighting their potential applicability in the treatment of AD [316]. In another study, aqueous leaf extract from *Lespedeza juncea (*Chinese lespedeza) was used to create quasi-spherical AgNPs that were 50 nm or smaller in size. The findings indicated that these NPs exhibited significant inhibitory effects on all the enzymes examined. For example, the IC₅₀ values for AChE and butyrylcholinesterase (BChE) were determined to be 9.3 ± 1.3 and 32.65 ± 1.9 μg/mL μg/mL, respectively. Regarding BChE, the AgNPs exhibited even more potency in their action compared to the standard inhibitor galantamine hydrobromide (IC_50_ = 40.83 ± 0.9 μg/mL). Given that the enzymes studied play a crucial role in AD, these findings are interesting, as inhibitors of AD are effectively employed in disease management [317].

Studies have shown that oleic acid’s phenolic derivatives serve as efficient reductants in the AgNP synthesis process [304]. When creating AgNPs for use in streptozotocin-induced AD rat models, *Nepenthes khasiana* leaf extract was utilized as a reducing and capping agent. The capping of *N. khasiana* leave extract polyphenols onto the surface of AgNPs was verified by Fourier Transform Infrared Spectroscopy (FT-IR) studies, which was essential to prevent deficiencies in spatial memory and recognition [304, 318]. In another study, the nanosilver aqueous extracts of *L. cocineus* and *M. lutea* showed high anti-ACHE [316]. The annual plant *Kickxia elatine* is used in conventional medicine as a sedative, general tonic, and wound-healing agent, as well as for lacrimation and bleeding. *Kickxia elatine* phyto-chemical constituents have been analyzed in only a few species worldwide, leading to the isolation of conferring good anti-glycation activity (inhibition of *α*-glycosidase), iridoid, flavonoids, and glycosides. Four types of flavonoids (acetylpectolinarin, pectolinarin, glycosides, and flavone) were extracted from *Kickxia elatine*, suggesting that it has strong antioxidant properties and can prevent the development of heart disease, diabetes, cancer, and certain other disorders like AD and dementia [37, 319]. For example, the green synthesis of AgNPs was investigated using the plant *Kickxia elatine* extract by Huda et al., These green-synthesized AgNPs showed significant anti-AChE activity at 175 μg/mL. *Kickxia elatine* and AgNPs can be regarded as an inhibitor of rats’ brain AChE, according to the study’s researchers [37]. Furthermore, the synthesis of biogenically synthesized AgNPs and AuNPs of *Acacia auriculiformis* leaves can be used as a cheaper alternative for AD treatment [320]. Small-size, stable, and potent green-synthesized Ag/Au bimetallic NPs (Ag/Au BNPs) from *Heliotropium eichwaldi L. (HE)* extracts were showed activity of anti-AChE, making them an important treatment for AD [321]. The synthesized AgNPs from *Scoparia dulcis* were evaluated for their in vitro inhibitory activity of AChE using an Ellman’s test; the IC50 value was 149 μg/ml. According to the authors, the bioactive compounds found in the plant extracts reducing Ag ions and capping the NPs are responsible for the green-synthesized AgNPs’ potential antioxidant action [38].Also, the green-synthesized AgNPs from *Millettia pinnata* flowers extract showed an excellent inhibitory efficacy against AChE [322]. Another study found that green synthesized ZnO NPs using methanolic extracts of *Sabal black Burniana* were more promising in vitro AChE inhibitory activity than DZP in AD. Molecular docking studies confirmed it even more. Inside the human AChE’s active site, the top-scoring flavonoids produced from *Sabal* demonstrated comparable binding mode with galantamine (i.e., co-crystallized ligand), and this could offer a molecular explanation for the anti-AChE activity of extracts from *Sabal* [315]. ZnO NPs made from *Aquilegia pubiflora* aqueous leaves extract also demonstrated outstanding inhibitory potential against the AD-related enzyme AChE (IC50: 102 μg/mL) [323]. The synthesized metal-based NPs from plant extracts with anti-AChE activities for the treatment of AD are listed in Table 3.

**Table 3:** The synthesized metal nanoparticles from plant extracts with anti-acetylcholinesterase activities for the treatment of AD

NPs	Plants	Plant tissues for extraction	Size (nm)	Shape	IC_50_ values (μg·mL^−1^)		Ref
Ag	*Kickxia elatine (KE)*	Whole plant	42.47	Spherical	117.76 ± 5.21	2023	[37]
Ag	*Scoparia dulcis L*	Leaves	21	Spherical	149	2023	[38]
Ag/Au BNPs	*Heliotropium eichwaldi L*	Whole plant	14	Rod	71.2 ± 0.22	2023	[321]
Ag	*Lampranthus coccineus* and *Malephora lutea*	Aerial parts	12.86–28.19	Spherical	Not available	2019	[316]
Ag	*Aquilegia pubiflora*	Leaves	19	Spherical, elliptical and cubic	71.38 ± 1.75	2021	[324]
Ag	*Millettia pinnata*	Flower	16–38	spherical	334.9 ± 1.52 mg/ml	2017	[322]
Au	*Terminalia arjuna*	Bark	20–50	Spherical and triangular	4.25 ± 0.02	2018	[33]
Au	*Saussurea costus*	Whole plant	19	Spherical	41.33	2024	[325]
ZnO	*Sabal blackburniana*	Fruits, pollen grains, and leaves	2.23–49.56	Spherical	Green synthesized ZnO NPs using pollen grains, fruits and leaves extracts; 63.78 ± 1.04651, 81.985 ± 3.075 and 117.95 ± 6.858 ng/ml respectively	2021	[315]
ZnO	*Aquilegia pubiflora*	Leaves	34.23	Spherical or elliptical	102	2021	[326]
ZnO	*Moringa oleifera*	Leaves	10–25	Hexagonal	Not available	2021	[39]
Fe_3_O_4_	*Selaginella doederleinii*	Whole plant	30	Spherical	0.73 ± 0.009 μmol/L	2022	[40]

The half-maximal inhibition concentration (IC50) needed to produce 50% inhibition of the enzymatic reaction (inhibition of the hydrolysis of acetylcholine) was determined. An aqueous extract of the entire *Convolvulus pluricaulis* plant impregnated with synthetic Fe_3_O_4_ was found to improve cognition in interoceptive and exteroceptive models by blocking the cholinergic receptor of AChE. Fe_3_O_4_ may therefore be advantageous in AD. The AChE catalytic activity was evaluated in HepG2 cell lines following treatment with Fe_2_O_3_ NPs in this investigation, and the outcomes validated Fe_2_O_3_ NPs inhibitory effects on AChE activity in vivo [327]. In addition, the conjugation of anthocyanin with AuNPs can improve the neuroprotective qualities of dietary polyphenolic compounds such as anthocyanin [328]. Suganthy et al., for instance, documented the neuroprotective properties of biogenic AuNPs produced using *Terminalia arjuna*. Their findings demonstrated the biogenic AuNPs biocompatibility and strong neuroprotective properties [33]. At very low concentrations, the biogenic AuNPs effectively inhibited the ChE enzymes, decreased the Aβ fibrillation process, and destabilized the mature fibrils [33, 130]. *Saussurea costus* is rich in numerous aromatic functional groups containing polyphenolic compounds, which possess a wide range of biological properties. In this regard, a study focused on utilizing *Saussurea costus* extract for the green synthesis of Au-NPs. It was observed that the methanolic *Saussurea costus* extract had the highest concentration of major phyto-constituents, and demonstrated the in vitro anti-AD Activity [325]. In addition, some natural substances (huperzine A, QT, GAL, vinpocetine, and etc.), and extracts of various plants (as mentioned in the previous sections) are used to treatment of AD. These plant preparations produce fewer undesirable effects and the same effectiveness as the classic therapy, or these preparations are used as a supplement to the classic therapy [329, 330]. For example, antioxidant agents such as QT slow down AD progression, but the usage of this flavonoid has limitations because of its low bioavailability [331]. However, NPs greatly increase the therapeutic efficacy of bioactive compounds by preventing their degradation and enhancing their cellular uptake [332]. To boost the uptake and bioavailability of loaded compounds, for instance, an uptake enhancer might be applied to the surface of NPs [333].

A Cur-based nanodelivery system, for instance, dramatically enhances the stability and water solubility of Cur via combining it with polyvinylpyrrolidone and modification using AuNPs (PVP–C–AuNP). This nanosystem exhibits exceptional long-term stability, controlled release characteristics, and a large drug loading capacity. Studies employing nuclear magnetic resonance and transmission electron microscopy have confirmed that PVP-C-AuNP inhibits Aβ1-16 aggregation and shows that it can break apart preformed aggregates [334].

In a different research, Liu et al. designed an AuPd core–shell structure coated with QT modified polysorbate 80 (P-80) to remove Aβ through the autophagy pathway in order to cure AD. This system combines a number of innovative advantages: a polysorbate 80 coating significantly increases the efficiency of BBB penetration by blocking P-glycoprotein efflux and adsorbing ApoE to form low-density lipoprotein complexes; the AuPd alloy core has peroxidase-like activity, which efficiently scavenges free radicals and reduces oxidative stress, offering a supplementary antioxidant mechanism to QT; and its concave cubic structure gives the NPs superior stability. When QT is administered to the brain site, it synergistically induces autophagy through enhancing autophagosome-lysosome fusion, which speeds up the clearance of Aβ. A two-pronged attack on Aβ pathology and related oxidative harm or neuroinflammation is produced by the functional synergy between the intrinsic antioxidant (radical scavenging) capability of the NPs and QT’s pro-autophagic activity. QT@P-80@AuPd significantly decreased Aβ aggregation, suppressed the activity of caspase-3, and restored the neuronal function in SH-SY5Y cells and APP/PS1 mouse models [335].

To sum up, the combination of phytochemicals with nanodelivery technologies has created a new avenue for the treatment of AD. Nanotechnology has effectively conquered the bioavailability and BBB penetration challenges of natural compounds, exhibiting significant neuroprotective potential. This has been achieved through specific targeted delivery, improved pharmacokinetics (enhancing stability and bioavailability), and, most importantly, multi-mechanistic synergistic regulation (which often includes the inherent therapeutic properties of the nanocarriers themselves, working in harmony with the loaded phytochemicals) [336].

#### Green MNPs crossing the blood–brain barrier

Because MNPs can be produced using environmentally friendly techniques while retaining their distinct physicochemical characteristics, they are a great option for treating NDDs like AD, where accurate and safe therapeutic agent delivery is essential. Furthermore, green synthesized MNPs' lower toxicity and environmental effect match the rising desire for more ethical and sustainable medical care [41, 315]. All things considered, the green synthesized MNPs present a viable way to get around the BBB. Compared with conventional drug formulations, these MNPs, which are derived from plant sources, have special biological and physicochemical characteristics that allow them to pass the BBB more effectively [337].

Many factors affect the delivery of nanodrug systems to the brain during the nanocarrier preparation process. Their permeability and targeting efficiency can be improved by modifying and adjusting specific factors, including ligand characteristics, particle size, and nanocarrier morphology [338]. The size of NPs is a determinant of their capacity to cross the BBB. An NP has a higher chance of crossing the BBB if it is smaller [150, 151]. The kidneys are likely to eliminate NPs smaller than 10 nm, though, and this must be acknowledged [153]. Owing to their inherent instability, metal particles formed in the aqueous phase have a tendency to aggregate in order to reduce the total surface energy. Some metals also act as nuclei on which other metals can grow. By suppressing this agglomeration, which may be brought on by attractive van der Waals forces between crystals, the ultimate particle size at the nanometric scale can be kept to a minimum. The plant extract improves the NP size control by acting as a dispersing and reducing agent to separate metal ions from one another [339, 340].

In addition, green synthesized MNPs can improve drug transport, stability, and specificity by utilizing their high surface area, biocompatibility, and potential for functionalization with different therapeutic agents. This will improve the results of AD treatment [337]. It is reported that when paired with surface modifications like PEGylation and functionalization with therapeutic molecules, green synthesized MNPs provide a very efficient way to get medications over the BBB and into the afflicted parts of the brain [341].

Therefore, the intrinsic properties of NPs derived from medicinal plants enable successful BBB traversal, allowing for targeted delivery to the brain and the modification of particular molecular pathways implicated in neurodegeneration [342].

## Removal and retention of the capping of biogenic metal nanoparticle

Extracellular proteins, amino acids, enzymes, and secondary metabolites are among the biomolecules and compounds that make up the capping of biogenic NPs. These substances are obtained from the metabolism of the fungus or other biological agents [343–345]. The biogenic capping agents function as binding molecules or stabilizers that change the surface chemistry and biological activity, stop agglomeration and steric hindrance, and maintain the interaction of NPs in the preparation medium. This capping, which provides stability, is typically present in MNPs made by biogenic synthesis; however, other materials might be added to give certain activities that enhance the NPs’ effects [345–347]. According to a study by Shaheen and Ahmad, phytochemicals minimize NP aggregation in addition to aiding in the reduction process [348]. In a similar vein, Sahayaraj et al., demonstrated that secondary metabolites in the plant extract not only improve the stability and dispersity of the NPs but also contribute as fabrication agents [349]. To gather information about the biomolecules that may be involved in capping and metal ion reduction have been identified through the use of FT-IR analyses [350, 351]. According to a study (some of the authors of this paper), the biomolecules’ O–H and C = C functional groups in *Daphne mezereum’s* aqueous leaf extract may have contributed to the stabilization and reduction of the iron NPs [352]. Furthermore, according to an FT-IR analysis of IONPs prepared using aqueous leaf extract from *Teucrium polium*, some of the authors of this paper demonstrated in another study that C = C ring stretching is the revealing of AgNPs functionalization with organic molecules [353]. Additionally, an FT-IR analysis of Fe_2_O_3_ NPs produced using *taranjabin* (camelthorn or persian manna) aqueous extract suggests that C = O and C-O stretching vibrations as well as OH groups of alcohols and phenols may have participated in these processes (prior work by some of the authors of the current article) [354]. In another study, Kohbanani et al., (some of the authors of this paper) used plant extract from *Ducrosia anethifolia* as a reducing and stabilizing agent to green synthesis Ag–Fe core–shell NPs. The functional groups on the AgNPs surface were examined using FTIR analysis. The findings suggested that these processes might have involved amide groups, C–OH bond stretching, and C-H bending [16]. Furthermore, it was demonstrated in a different study by Kohbanani et al., that *Plantago* major Leaf Extract is a significant capping agent. FT-IR analysis revealed that the band at around 2059 cm^−1^ is due to the O–H of carboxylic acids IONPs. Stretching vibrations of C = C ring were assigned to the moderateintensity band at 1635 cm^−1^. These peaks demonstrated both the biological coating on the surface of NPs and the green synthesis of IONPs (prior work by some of the authors of the current article) [24]. Biomolecules with hydroxyl, amine, and carboxyl functional groups were implicated in the reduction of Au ions, according to FT-IR analysis of Au NPs green synthesized using *Suaeda monoica* leaf extract [355]. An −OH functional group was probably involved in the synthesis of Au NPs, according to the corresponding FT-IR spectra, which were obtained using chlorogenic acid as a reductant [351]. Furthermore, compounds with amide, carboxyl, alkyne, and hydroxyl groups of the monoterpenoids, sesquiterpenes, and phytols may have taken part in these processes, according to an FT-IR analysis of AgNPs made with bark extract from *Acacia mearnsii* [350]. The existence of alkene, phenol, alkene, alkynes, aliphatic amines, amide, proteins, carboxyl, polyhydroxy, lipids, halo compounds, etc. was confirmed by FT-IR analysis at the corresponding peaks of *Kickxia elatine* and AgNPs, according to a study by Huda et al. In the reduction of AgNPs, the alcoholic and phenolic compounds may act as stabilizing mediators. The carbonyl compounds of proteins attribute to the stabilization of AgNPs by capping them [37]. The FT-IR analysis of AgNPs generated using *Neem (Azadirachta indica)* leave extract was used to observe the functional groups such as phenol, alcohol, methoxy, carbonyl, amide, and ethylene attached to stabilize the NPs [356]. Furthermore, analysis of AgNPs green synthesized with leaves extract of *Withania somnifera* using high performance liquid chromatography (HPLC) showed that a number of phenolic compounds in the extract were specifically trapped in the AgNPs. Furthermore, a major compound that was present in the NPs but absent from the original extract may be a derivative that arises from the interaction of certain withanolide derivatives with Ag ions [109, 357].

In the light of above discussion, the majority of research has demonstrated that the green synthesized NPs which are used must be calcined at ultra high temperatures in a blast furnace to achieve the highest degree of crystalline morphology. This will undoubtedly result in a pure-phase crystalline structure, but the phytochemicals attached to the surface as capping agents will break down [109]. Furthermore, numerous investigations have demonstrated that centrifugation is a simple method for removing capping agents. According to Guilger et al., the capping was taken off of half of the volume of the samples in order to look into possible differences between capped and uncapped AgNPs [345]. The NPs dispersions were centrifuged in this investigation, followed by a resuspension and boiling of the pellets in 1% sodium dodecyl sulfate (SDS), and another centrifugation was performed. Before subsequent enzyme and protein analyses, the supernatants containing the capping were kept at −20  C. The pellets were boiled in 60 mM Tris–HCl pH 6.8, and then they were dialyzed using a Visking MWCO 12–14 kDa membrane to produce NPs without capping. According to the study’s findings, capping was crucial in regulating the size of the NPs and enhancing their biological activity against *S. sclerotiorum* [345].

In summary, since the capping of biogenic NPs is what gives them their colloidal stability, removing it can change their properties. Green synthesized NPs are frequently capped with secondary metabolites, as was previously mentioned. In addition to serving as stabilizing agents or capping agents for the green synthesized NPs, these compounds may also improve the NPs’ bioactivities [345, 358].

### The effect of secondary metabolites on the size, shape and stability of metal nanoparticles compared to chemical method

While the majority of research has not examined the green synthesized NPs for the presence of extract-derived compounds, a number of studies have shown that proteins, secondary metabolites, and sugars conjugate in these NPs. The bioactivities (catalytic, cytotoxic, antioxidant, anti-microbial, and etc.) of green synthesized NPs have been reported numerous times, but very few studies have contrasted these activities with those of their chemically synthesized counterparts [109]. According to some studies, tiny NPs are produced by the stronger chemical reducing agents such as alkali metal borohydrides (AMB) while extracts from plants behave as poor reducing agents, tend to nucleate particles much more slowly and generate big NPs and polydisperse products that may require post-processing [359].

On the contrary, some articles have reported that, when compared with conventional chemical methods, green synthesis frequently produces NPs with smaller and more uniform sizes. For instance, Fe_3_O_4_ NPs produced using the green synthesis method (using the biocomponents of Hibiscus rosa-sinensis) have particle sizes of 2–80 nm, which is significantly smaller than the 87–400 nm particles produced using the wet chemical method. In green synthesis, the phytochemicals in the leaf extract serve as both a reducing and a capping agent, whereas these NPs made by chemical means are unstable and prone to aggregation [360]. Similarly, in another study, the size of IONPs was analysed by the HR-SEM and the mean size of the particles was 20.9 nm and 42.3 nm for green synthesized IONPs and chemical synthesized IONPs, respectively [361]. According to the Dowlath et al., the *C. halicacabum* ethanolic extract contains a large range of metabolites that aid in stabilizing and reducing iron ions without aggregating them. Whereas, in chemical synthesis, the produced NPs have a tendency to aggregate more quickly, making it difficult to keep the NPs size [361]. In addition, Kouvaris et al., [362] and Kummara et al., [363] reported that the size of NPs produced by chemical methods is larger than that of NPs from plant extracts. This is due to the plant extract's stabilizing agents and surfactants, which cover the particles and provide stability without agglomeration [361–364].

Considering the fact that some researchers have reported that green synthesized MNPs are smaller than chemical-synthesized MNPs and vice versa. We will discuss the factors relevant in controlling the size of MNPs in the following.

By varying the initial concentration of the substrate, the ratio of solvent to non-solvent, the stirring speed, the titration speed, and other variables like temperature and pH, the particle size can be controlled. Furthermore, the use of various capping agents can regulate not only the material's morphology, aggregation, and particle size but also the stability of nanostructures throughout time. Additionally, the kind of capping agent interaction with the NPs’ surface affects the nanostructure that is formed [365, 366].

In addition, reduction kinetics are crucial for determining the product’s identification in NPs syntheses. Changing concentrations can help to change reaction kinetics, but the reducing agent can also have a significant impact on the reduction potential [367]. Strong reducing agents such as AMB are favoured for their ability to produce tiny NPs [368, 369]. Although higher temperatures may be sufficient in some cases, it is not clear how to make up for the green reducing agents’ decreased reduction potential [370].

The following discusses the ideal settings for the green synthesis of MNPS, including temperature, pH, and the ratio of plant extract to metal ions; this can help standardize procedures and improve the reproducibility of findings in subsequent research.

*Reaction temperature:* Regarding the impact of reaction temperature, the majority of findings showed that as temperature rises, green NPs get smaller [367]. For example, Fayaz et al., showed that the size of green NPs decreases with increasing temperature whereas the size of these NPs rises with lowering reaction temperature [371]. In a different study, the impact of synthesis temperature on AgNPs growth was examined using *Vitex agnus-castus* extract. The temperature range was 40–80 °C with constant stirring, and the creation of NPs was tracked using the SPR band at 420 nm in UV–vis spectra. Higher temperatures (50–80 °C) enhanced SPR absorption and accelerated NPs growth, with the quickest production occurring at 80 °C. At 40 °C, smaller, less stable NPs (< 1 nm) formed. Because bigger phytochemicals that form the organic particle coating require higher temperatures to synthesize, the study found that NPs creation is very temperature-dependent, with optimal synthesis taking place between 60 and 80 °C [372]. Researchers also found that when the reaction temperature was raised from 25 °C to 60 °C, the size of the green-synthesized AgNPs decreased from 35 to 10 nm [372]. Using *olive* leave extract, Khalil et al., produced AgNPs. They found that raising the reaction temperature caused the Ag^+^ ions to reduce quickly and the subsequent homogeneous nucleation of Ag nuclei results in the formation of small AgNPs. As a result, it is widely and generally accepted that high temperature is conducive to nucleation while low temperature is conducive to growth in the field of wet chemical AgNPs synthesis [367, 373].

To this end, Jon and et al., carried out a study that used extract from *Zea mays* in the environmentally friendly synthesis of Au NPs. The UV–vis spectrum in this study showed prominent peaks that grew narrower and more intense as the temperature rose from 30 to 75 °C. This suggested that a rise in nucleation caused the size range of AuNPs to decrease [374]. The plant's seeds, roots, stems, flowers, and leaves can all be used to make plant extracts. Nevertheless, the extracts produced by these distinct plant parts have different compositions, which affect the size and characteristics of the resulting NPs [375].

*Precursor concentration and extract-to-metal ratio:* According to some studies, the type and concentration of secondary metabolites can affect the MNPs’ ultimate shape and size. For instance, the shape of Au NPs made with *Cinnamomum zeylanicum* leaf broth varied according to the extract's concentration. Au NPs of prism-shaped were the predominant form at lower extract concentrations, while spherical NPs predominated at higher concentrations [376]. Therefore, in synthesis processes, the molar ratios of reactants are crucial variables that affect the NP size. Perumal Karthiga showed how altering the reactant concentration can systematically change the shape of silver nanocrystals green synthesized with silver nitrate and citrus leaf extract. According to the authors, spherical NPs were produced with an AgNO3: citric acid ratio of 1:4 (vol:vol). Nevertheless, it was also shown that the size of AgNP particles increased when bio-organics were produced from plant extract. It was observed that precursors at a greater molar ratio significantly influenced the morphology of the NPs, even though there was no clear correlation between the precursor concentrations and the nanocrystal's structure [377]. In general, controlled synthesis leads to NPs with specific functionalities, enhancing their applications in fields like medicine and biotechnology. For instance, increasing the concentration of L-cysteine during AgNPs synthesis resulted in a decrease in average particle size from 17 to 3 nm [378]. Compounds such as flavonoids, alkaloids, and phenolics are effective in reducing metal ions to NPs, with their concentration directly affecting particle size [377]. For instance, research indicates that extracts with a higher phenolic content typically result in smaller NPs, perhaps as a result of their potent binding and ability to impede particle growth [379]. For instance, a study by Saputra et al., uses environmentally friendly leaves extract from *Oil palm* (Elaeis guineensis Jacq: EGLE), *Kapok* (Ceiba pentandra: CPLE), and *Mango* (Mangifera indica: MILE) trees to examine the impact of the extracts’ secondary metabolites on the synthesized ZnO NPs [379]. The findings demonstrated that, in comparison to ZnO_CPLE (94.5 nm) and ZnO_MILE (74.9 nm), ZnO_EGLE, which contained both saponins and phenols, displayed the smallest and most uniform NPs (43.3 nm). These results were corroborated by TEM analysis, which demonstrated the critical function of saponins and phenols as capping agents in regulating particle size and agglomeration [379].

As previously mentioned, a variety of secondary metabolites, including terpenoids, polyphenols, alkaloids, and phenolic acids, are present in various plant extracts, which serve as stabilizing and reducing agents during the synthesis of MNPs [368]. In addition to reducing metal ions, the plant metabolites also prevent agglomeration, limit the size of the NPs, and cap them [380].

*pH:* Studies have demonstrated that a basic environment is more conducive to the synthesis of smaller-sized NPs than an acidic solution [381]. The work conducted by Qian et al., provides an excellent example of how pH affects the environmentally friendly production of metallic nanoparticles, where they used several pH ranges for comparison and *Penicillium oxalicum* for the production of AgNPs. They have determined that NPs with a smaller size distribution and a quicker synthesis time are produced by alkaline pH. These features show enhanced stability as a result of the anions' electrostatic repulsion [382]. Kowshik examined the green-synthesized NPs' pH stability. It was found that particle aggregation resulted from the nanocrystallites' acidification from pH 7 to 6 [383]. In a different research, the quantity of AgNPs produced from *Cinnamon zeylanicum bark* extract rose as bark extract concentrations increased over time and at higher pH levels (pH 5 and above). Moreover, nanocrystals precipitated out of the solution at pH levels lower than 6 [384].

Briefly, numerous studies have documented that altering physiochemical parameters during synthesis, such as pH, temperature, and plant extract concentration, can control the shape and size of NPs [375, 380]. Which, this manipulation is also possible with the chemical method.

Therefore, the green or the chemical method can synthesize MNPs with smaller size only if the effective conditions in the synthesis of MNPs are identical in both methods. Furthermore, it is necessary to consider that different plants show various compounds and concentrations of reducing agents, which influence the size, shape, and stability of synthesized MNPs. As explained above, different concentrations of *Cinnamomum zeylanicum* leaf extract resulted in the production of AuNPs with different shapes. Or, for example, to achieve smaller sizes of NPs synthesized by the Green method, it is better to choose plants that have higher phenolic compounds. Finally, the properties of MNPs must be matched to their functional purpose in order to select the appropriate method for synthesizing MNPs. For example, as mentioned in Sect. “Blood–brain barrier: different pathways and factors affecting the crossing of MNPs”, NPs of different sizes choose different methods to cross the BBB.

## Green synthesis challenges and alternative solutions

Despite promoting biocompatibility and reducing the use of harmful substances, green synthesis is frequently constrained by certain issues. Lack of uniformity in biological sources, process control issues, and difficulties with large-scale production are the main causes of these limitations. Variability in biological extracts, for instance, is a significant problem. The composition of phytochemicals may change due to environmental factors or seasonal variations. Reproducibility may be impacted by this. In addition, scaling up is not always simple. The stability and concentration of plant extracts vary. Particle size distribution is still hard to control [385, 386]. There may be differences in the composition and activity of plant extracts between batches because of the inherent variability in plant material and extraction techniques. This may result in irregularities in the synthesis procedure and the characteristics of the produced NPs [387].

Phytochemical and pharmacokinetic limitations impede the therapeutic application in addition to synthesis-related difficulties. For example, certain herbal substances' limited absorption has made it difficult to use them for therapeutic purposes. For example, when used to treat AD, the polyphenol curcumin shows limited absorption and bioavailability [342, 388]. Finding the right dosage to create efficient formulations to maintain bioavailability and figuring out when to take herbal medications, such green synthesized MNPs, are two issues that still need to be resolved in the clinical usage of these medications against AD [389].

Comprehensive evaluations of safety and efficacy are required, as bioactive phytochemicals which are present on NP surfaces may lead to unforeseen biological interactions.

In addition, there is not adequate information about the toxicity of green-synthesized NPs. The NPs may exhibit detrimental effects despite their natural synthesis, contingent on exposure, dose, and tissue accumulation [390]. Therefore, thorough toxicity studies are essential to resolve the safety issues related to green-synthesized NPs. Future studies must methodically examine these NPs' immediate and long-term impacts on the environment and human health. Evaluating possible immunogenicity, genotoxicity, cytotoxicity, and biodegradability is part of this [391]. The safe and efficient application of green-synthesized NPs in clinical and industrial settings will be ensured by regulatory frameworks informed by strong toxicity data [386].

Another significant barrier from a manufacturing standpoint is the absence of standardized procedures for green synthesis. It is difficult to compare results and establish best practices because different studies frequently use different conditions and methods [386]. In order to implement green nanotechnology in clinical settings, a number of clinical and regulatory requirements must be met. Phase I–III clinical studies are required before clinical trials and regulatory approval in order to ascertain dose, effectiveness, safety, and therapeutic index. Compiling thorough characterization, production, and safety data is another requirement for regulatory submissions (like Investigational New Drug (IND) or New Drug Application (NDA) applications) [392, 393]. The creation of global guidelines is another essential stage in the clinical acceptability process. In this regard, it is essential that international regulatory bodies collaborate to develop consensus-based frameworks for assessing green NPs that incorporate criteria specific to green NPs in International Organization for Standardization (ISO) and Organisation for Economic Co-operation and Development (OECD) recommendations [392, 394].

## Applying nanotechnology and artificial intelligence to treat Alzheimer’s disease

Artificial intelligence (AI) technologies have been included due to the difficulty in analyzing data, the volume of data, and the time-consuming, expensive, and complex nature of laboratory procedures [395]. The integration of nanotechnology and AI is paving the way for a new generation of intelligent systems aimed at the diagnosis, monitoring, and treatment of NDDs. Each domain provides unique strengths: Nanomedicine offers high molecular precision, while AI excels in data-driven adaptability [396]. By leveraging algorithms capable of processing extensive datasets, AI facilitates decision-making, optimization, and uncovering in the development of green NPs [397–399]. In addition, AI may be used to optimize drug delivery nanosystem design, characterization, and manufacturing, which will speed up the creation of safe and efficient treatment options [395]. For instance, computational pharmaceutics, which aims to improve drug delivery techniques using multiscale modeling methodologies, was developed as a result of the pharmaceutical industry's integration of AI and big data. Computational pharmaceutics analyzes massive datasets and forecasts drug performance using AI algorithms and machine learning (ML) techniques [395, 400]. The estimates indicate that incorporating AI can potentially improve efficiency, lower expenses, and accelerate drug development [401].

However, the lack of functionally validated targets and experimentally confirmed bioactive compounds makes AI-based drug development for AD extremely difficult. Structure-based medication discovery is additionally hampered by the high structural abnormalities of recognized pathologic targets, such as tau and amyloid precursor protein. Using pre-training models on huge datasets, like ImageMol, is one method of addressing these issues [402]. Active learning is a further option that allows for iterative model updates while labeled data is limited [403].It is anticipated that future studies would examine AI techniques, namely task analysis, with an emphasis on utilizing deep neural networks and combining multimodal data [404–406].

The next advancement in the field is personalized nanomedicine, where AI integrates multi-omics profiles, imaging biomarkers, and longitudinal clinical data to regulate interventions at the molecular level. The advanced models of machine learning (ML) are now capable of simulating disease trajectories and therapeutic responses in silico, which helps to optimize NPs formulations, dosing regimens, and payload selection for individual patients. For example, in patients with high oxidative stress signatures, AI might modify BBB-targeting ligands in response to evolving neurovascular profiles or recommend ROS-responsive drug delivery systems [396].

Furthermore, the use of organoid models, AI-based carrier design, and multi-omics analysis technologies is promising for improving personalized nanotherapeutics (especially those that make use of specific functional synergies between cargo and carriers) and further clarifying the molecular networks of AD [336].

These methods could aid in the discovery of new risk factors, such as environmental factors that affect the onset of AD, improve the accuracy of disease progression predictions, and aid in the creation of successful preventative measures. Long-term, AI-driven advancements might change the way AD is managed from a progressive, incurable state to one that is easier to control and perhaps reversible. This can result in better long-term care, rehabilitation, and healthcare options [404–406].

## Conclusion and future prospects

MNPs have a wide range of therapeutic applications in neurodegenerative diseases such as AD, which are constantly expanding. Many studies have been recently conducted on plants as potential sources of producing MNPs from their inorganic metal ions. Metabolites like polyphenols, terpenoids, proteins, and flavonoids are frequently found in plant extracts and bioextracts. In addition to serving as reducing agents for metal ion reduction, these biomolecules also serve as capping agents, which help to reduce NP agglomeration and control morphology. They also help to protect and stabilize the NPs, which improves their biological potential.

Plant metabolites can be integrated with MNPs to improve their therapeutic efficacy against AD. Secondary metabolites give a range of renewable options of capping/stabilizing/ agents that can be produced locally and at a minimal cost.

In order to specify the structural relationship of NPs, additional analysis of the stabilizing, capping, or reducing agents involved in the green synthesis is required. To determine whether there is a relationship between the particular chemicals and the NPs produced, a high-throughput examination of plant extracts containing a variety of metal ions might be helpful. The use of metal ions or NPs to trap compounds that are costly or challenging to purify using existing methods would be extremely beneficial.

In order to create green-synthesized NPs with sticky capping agents and functionalized surfaces for better biological applications, synthesis techniques must be optimized. Factors like NPs surface charge, size, capping agents, and coating have a significant impact on safety, stability, BBB permeability, and efficacy because the translational potential of MNPs synthesized using plant extracts is heavily influenced by their plant metabolite composition, synthesis techniques, and physicochemical characteristics.

Utilizing plant metabolites for green synthesis has benefits in enhancing biocompatibility, decreasing aggregation, and regulating particle homogeneity—all of which are critical for clinical applications and production scaling. Researchers can improve MNPs for therapeutic delivery, reduce possible toxicity, and increase reproducibility by methodically taking these factors into account. This will open the door to more potent and translationally relevant treatments for AD.

However, to evaluate their efficacy in the metabolic system, MNPs active compounds must be investigated for in vivo activity in both animal and human models. Future clinical trials with bigger sample numbers should also investigate the function and underlying processes of different medicinal herbs in the management of AD. To address the concerns of kinetics, reproducibility, uniformity, stability, and yielding in particular, more research is needed for large-scale production.

Additionally, scientists may detect any dangers and difficulties associated with DDSs in the early stages of development by utilizing the right AI technologies. This allows them to make proactive adjustments and modifications to reduce risks and improve drug performance.

## Data Availability

No datasets were generated or analysed during the current study.

## Abbreviations

AD: Alzheimer’s disease
Ach: Acetylcholine
AChE: Acetylcholinesterase
AChEI: Acetylcholinesterase inhibition
GAL: Galantamine
NPs: Nanoparticles
MNPs: Metal nanoparticles
FDA: Food and Drug Administration
AuNPs: Gold nanoparticles
Aβ: Amyloid β
AgNPs: Silver nanoparticles
ROS: Reactive oxygen species
ZnO NPs: Zinc oxide NPs
IONPs: Iron oxide NPs
DHA: Docosahexaenoic acid
PUFA: Polyunsaturated fatty acid
APP: Amyloid precursor protein
PREN 1: Presenilin 1
APOE-4: Apolipoprotein E-4
NFTs: Neurofibrillary tangles
Tau: Tubulin associated unit
KD: Kilodalton
APP: Amyloid precursor proteins
sAPP: Soluble amyloid precursor protein
nAChRs: Nicotinic acetylcholine receptors
BuChE: Butyryl ChE
NMDA: N-methyl-d-aspartate
Au: Gold
Pt: Platinum
Ag: Silver
Ru: Ruthenium
Ni: Nickel
Ti: Titanium
Cu: Copper
Fe: Iron
Co: Cobalt
Zn: Zinc
RNS: Reactive nitrogen species
BBB: Blood–brain barrier
PPD: Passive paracellular diffusion
PTD: Passive transcellular diffusion
SCP: Solute carrier proteins
RMT: Receptor-mediated transcytosis
AMT: Adsorptive-mediated transcytosis
TJM: Tight junction modulation
PLGA: Poly (lactic-co-glycolic acid)
HA: Hyaluronic acid
NDDs: Neurodegenerative diseases
OS: Oxidative stress
TNF-α: Tumor necrosis factor
NO: Nitric oxide
HFD: High-fat diets
RA: Rosmarinic acid
FA: Ferulic acid
TCM: Traditional Chinese medicine
CA: Caffeic acid
CGA: Chlorogenic acid
GA: Gallic acid
QT: Quercetin
KMP: Kaempferol
Lu: Luteolin
EGCG: Epigallocatechin-3-O-gallate
CREB: Cyclic adenophosphate-response element binding protein
BDNF: Brain-derived neurotrophic factor
TLR4: Toll-like receptor 4
BB: Berberine
HAR: Harmine
HAL: Harmaline
RE: Reserpine
AJ: Ajmalicine
PAS: Peripheral anionic sites
BB: Berberine
BCHE: Butyrylcholinesterase
PVP–C–AuNP: Cur with polyvinylpyrrolidone and modifies it with AuNPs
P-80: Polysorbate 80
GbE: Ginkgo biloba extract
FT-IR: Fourier transform infrared spectroscopy
HE: Heliotropium eichwaldi L
KE: Kickxia elatine
QT-SPIONs: Superparamagnetic IONPs
HPLC: High performance liquid chromatography
SDS: Sodium dodecyl sulfate
AMB: Alkali metal borohydrides
AI: Artificial intelligence
ML: Machine learning
IND: Investigational new drug
NDA: New drug application
ISO: International organization for standardization
OECD: Organisation for economic co-operation and development
SERM: Selective estrogen receptor modulator
DDSs: Drug delivery systems
